# Predicting visual working memory with multimodal magnetic resonance imaging

**DOI:** 10.1002/hbm.25305

**Published:** 2020-12-05

**Authors:** Yu Xiao, Ying Lin, Junji Ma, Jiehui Qian, Zijun Ke, Liangfang Li, Yangyang Yi, Jinbo Zhang, Cam‐CAN, Zhengjia Dai

**Affiliations:** ^1^ Department of Psychology Sun Yat‐sen University Guangzhou China; ^2^ Cambridge Centre for Ageing and Neuroscience (Cam‐CAN) University of Cambridge and MRC Cognition and Brain Sciences Unit Cambridge UK

**Keywords:** fMRI, machine learning, MRI, multimodal imaging, working memory

## Abstract

The indispensability of visual working memory (VWM) in human daily life suggests its importance in higher cognitive functions and neurological diseases. However, despite the extensive research efforts, most findings on the neural basis of VWM are limited to a unimodal context (either structure or function) and have low generalization. To address the above issues, this study proposed the usage of multimodal neuroimaging in combination with machine learning to reveal the neural mechanism of VWM across a large cohort (*N* = 547). Specifically, multimodal magnetic resonance imaging features extracted from voxel‐wise amplitude of low‐frequency fluctuations, gray matter volume, and fractional anisotropy were used to build an individual VWM capacity prediction model through a machine learning pipeline, including the steps of feature selection, relevance vector regression, cross‐validation, and model fusion. The resulting model exhibited promising predictive performance on VWM (*r* = .402, *p* < .001), and identified features within the subcortical‐cerebellum network, default mode network, motor network, corpus callosum, anterior corona radiata, and external capsule as significant predictors. The main results were then compared with those obtained on emotional regulation and fluid intelligence using the same pipeline, confirming the specificity of our findings. Moreover, the main results maintained well under different cross‐validation regimes and preprocess strategies. These findings, while providing richer evidence for the importance of multimodality in understanding cognitive functions, offer a solid and general foundation for comprehensively understanding the VWM process from the top down.

## Graphical abstract

We used multimodal neuroimaging in combination with machine learning to reveal the neural mechanism of visual working memory (VWM) across a large cohort. The resulting model exhibited promising predictive performance on VWM (*r* = .402, *p* < .001), and identified features within the subcortical‐cerebellum network, default mode network, motor network, corpus callosum, anterior corona radiata, and external capsule as significant predictors. Our findings, while providing richer evidence for the importance of multimodality in understanding cognitive functions, offer a solid and general foundation for comprehensively understanding the VWM process from the top down.

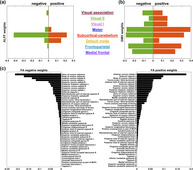

## 1 INTRODUCTION

Visual working memory (VWM) is the actively retaining and processing of visual information in aids of ongoing tasks during a short period of time. It is fundamental in human daily life for being indispensable from simple behaviors like direction identification and cash payment to more complex ones like web search and test taking. Besides, VWM is strongly correlated with cognitive ability (Luck & Vogel, 2013). It lays a ground foundation for human cognitive behaviors and provides a link between basic and higher cognitive functions. A number of mental diseases, including schizophrenia, Alzheimer's disease, and Parkinson's disease, have been found to accompany significant VWM capacity reduction (Gold et al., 2006; Lee et al., 2010; Leonard et al., 2013). In addition, the capacity of VWM has long been recognized to exhibit essential variation across individuals (Vogel, McCollough, & Machizawa, 2005). Such individual differences are stable and largely account for variation in higher cognitive functions (Luck & Vogel, 2013). Fukuda, Vogel, Mayr, and Awh (2010) reported that VWM accounted for 43% of individual differences in global fluid intelligence (FI), while Johnson et al. (2013) found VWM accounted for 46% of individual differences in the overall performance on a broad battery of cognitive tasks. Therefore, unraveling the neural basis of VWM at the individual level is of great importance for better understanding cognition in both cohorts of healthy people and patients with one or more of the aforementioned diseases.

Neuroimaging offers an efficient and in‐vivo method to explore the neural basis of VWM (Luck & Vogel, 2013). Biomarkers significantly correlated with VWM capacity were found from the function and structure of human brain, including the amplitude of low‐frequency fluctuations (ALFF) in the middle frontal gyrus and parietal lobules (Li, He, Wang, Hu, & Guo, 2017; Zou et al., 2013), the gray matter volume (GMV) of the dorsolateral frontal gyrus and hippocampus (Cannon et al., 2005; Guo et al., 2014; Strangman et al., 2010), and the fractional anisotropy (FA) in intraparietal sulcus and corpus callosum (Klingberg, 2006; Olesen, Nagy, Westerberg, & Klingberg, 2003; Takeuchi et al., 2010). However, these studies were all based on mono‐modality data. In fact, few studies have considered the possibility of using joint information from different modalities. Since structural magnetic resonance imaging (MRI) captures the fundamental brain morphology, diffusion MRI captures the white matter integrity, while functional MRI (fMRI) captures temporal fluctuation level of brain regions (Griffa et al., 2017), analyzing multimodal MRI that combines structural and functional information may allow us to reveal the neural mechanism of VWM in a more comprehensive and integrative manner. Until now, only small efforts have been paid to this theme. Tseng et al. (2017) combined task‐fMRI, hippocampi volume, and DWI to study visual recognition memory. However, using multimodal imaging data to comprehensively examine VWM remains to be elucidated.

Most of the existing unimodal MRI studies used the correlation analysis method to explore the patterns of neural basis of individual differences in VWM. Recently, machine learning methods have been found to be a surging tool to assist research in neuroscience (Davatzikos, 2019; Serra, Galdi, & Tagliaferri, 2018; Woo, Chang, Lindquist, & Wager, 2017). Compared with traditional correlation analysis methods, the merits of machine learning mainly lie in four aspects. First, it enables multivoxel pattern analyses, which facilitates inspection of the relationship between cognitive behavior and multiple voxels in one or more brain regions simultaneously. Second, it allows concrete and quantified backtracking on neural features about how they are related to the cognitive behavior in concern, which helps to identify the important neural traits. For example, the weight of a neural feature in the model built by machine learning methods can be used to measure the contribution of the neural feature to the behavior and/or its degree of correlation with the behavior. Third, it has a systematic way to relieve overfitting issues associated with large‐scale and high‐dimensional neuroimaging data. The resulting prediction model thus gains improved generality, suggesting that it can better pinpoint the actual relationship and has border implications (Dwyer, Falkai, & Koutsouleris, 2018). Last but not least, machine learning methods can be well‐tailored for neural basis examination at the individual level, offering promising opportunities to precision medical care, including personalized diagnostic, prognostic, and treatment for diseases. These merits have inspired a variety of novel applications of machine learning methods on imaging data, including but not limited to detecting imaging signature for various diseases and disorders (Nielsen, Barch, Petersen, Schlaggar, & Greene, 2020; Pellegrini et al., 2018), exploring imaging‐based biotypes that can assist diagnosis, prognosis and treatment of patients (Chand et al., 2020; Song, Yang, Sui, & Jiang, 2017), seeking neural patterns to explain individual differences in affect, cognition, and behavior (Cui & Gong, 2018; Mihalik et al., 2019; Scheinost et al., 2019), and offering brain fingerprints for individual identification (Finn et al., 2015; Wachinger, Golland, Kremen, Fischl, & Reuter, 2015). In the field of VWM, machine learning methods have also been applied to predict individual differences from imaging data since the last decade. Karch, Sander, von Oertzen, Brandmaier, and Werkle‐Bergner (2015) applied linear discriminant analysis to classify VWM loads using electroencephalography (EEG) data and found that person‐specific models lead to better discriminative performance. Majerus et al. (2016) applied support vector machine to classify VWM loads based on fMRI data and found that the dorsal attention network and sensory processing cortices could offer promising predictors. Girdher, Gupta, Jaswal, and Naik (2020) used seven different machine learning techniques to predict VWM responses using EEG data and found that the random forest and the neural network performed the best. However, in the aspect of decoding the neural mechanism of individual VWM capability, little effort has been made on utilizing the advantages of machine learning methods (Postle, 2015), especially in combination with multimodal neuroimaging data.

In this article, we combined three indicators from three different modalities, GMV, FA, and ALFF, to investigate the neural basis of VWM. The three indicators were selected for two reasons. First, they formed a comprehensive feature pool that can capture both the functional and structural aspects of human brain with fine granularity (i.e., all indicators can be obtained at the voxel level). In particular, ALFF measures the spontaneous fluctuations in brain gray matter and can reflect the functional activity of brain. GMV and FA quantify the volume of gray matter and the integrity of white matter, respectively, and thus offer descriptions of voxel‐wise attributes of brain structure. Second, as stated before, there has been abundant evidences supporting the strong associations of these three indicators with VWM. Specifically, it has been found that the ALFF values of the relevant brain regions (e.g., middle frontal gyrus and parietal lobules) may have distinct roles in VWM processes (Li et al., 2017; Xu & Chun, 2006, 2009; Zou et al., 2013). In addition, cortical and subcortical volumes have been shown to be related to VWM (Cannon et al., 2005; Guo et al., 2014; Machizawa, Driver, & Watanabe, 2020; Strangman et al., 2010), indicating that GMV would be a promising indicator. The integrity of white matter pathways (i.e., FA) has also been found critical for individual VWM processing (Golestani et al., 2014; Klingberg, 2006; Lazar, 2017; Olesen et al., 2003; Takeuchi et al., 2010). Based on the above three indicators, a machine learning pipeline that includes the steps of feature selection, relevance vector regression (RVR), cross‐validation, and model fusion was proposed to build a model that predicts individual VWM capacity from features extracted from the above three modalities. The RVR is a promising method for continuous variable prediction, especially when handling high‐dimensional data of a relatively small volume (Tipping, 2000, 2001). Besides, it can determine the hyperparameters in the prediction model automatically through a Bayesian learning strategy, which avoids the tediousness and unreliability induced by manual hyperparameter tuning. With the above efforts, we aim at achieving two goals: (a) build a generalized model to predict individual VWM capacity using multimodal MRI (R‐fMRI, T1, and DWI) data; (b) find discriminative brain regions contributive to individual VWM capacity for a more integrated understanding on the neural basis of individual VWM.

## 2 MATERIALS AND METHODS

### 2.1 Participants

All participants were from Stage 2 Cam‐CAN study (Shafto et al., 2014, www.cam-can.com). The R‐fMRI, T1 weighted image, DWI and VWM score data from 625 healthy adults were used in the current study. The data and the ethical approval for this study were obtained by the Cambridgeshire 2 (now East of England—Cambridge Central) Research Ethics Committee and written informed consent was obtained from each participant.

### 2.2 Data acquisition

All participants were scanned at the Medical Research Council Cognition and Brain Sciences Unit on a 3 T Siemens TIM Trio System with a 32‐channel head coil. The R‐fMRI was collected axially using a gradient‐echo echo‐planar imaging sequence. The image parameters were as follows: repetition time (TR)/echo time (TE) = 1,970 ms/30 ms; flip angle = 78°; field of view (FOV) = 192 × 192 mm^2^; time points = 261; slices = 32; direction = descend; thickness = 3.7 mm; voxel size = 3 × 3 × 4.44 mm^3^; gap = 0.74 mm. Participants were required to lie still with their eyes closed and remain awake. The scan lasted for 8 min and 40 s. T1‐weighted structural Magnetization Prepared RApid Gradient Echo (MPRAGE) 3D images were acquired using the following parameters: TR/TE = 2,250 ms/2.99 ms; inversion time (TI) = 900 ms; flip angle = 9°; FOV = 256 × 240 × 192 mm^3^; voxel size = 1 × 1 × 1 mm^3^; and GRAPPA acceleration factor = 2. The acquisition lasted for 4 min and 32 s. The twice‐refocused spin‐echo axial DWI were acquired using following parameters: 30 diffusion gradient directions for each of two *b*‐values: 1,000 and 2,000 s/mm^2^, along with three images acquired using b‐value of zero; TR/TE = 9,100/104 ms; voxel size = 2 × 2 × 2 mm^3^; FOV = 192 × 192 mm^2^; slices = 66; number of averages = 1. This acquisition lasted for 10 min and 2 s.

All the participants finished a modified version of a previous experiment (Zhang & Luck, 2008) that measured VWM ability. On each trial of the experiment, a participant was presented with one to four colored disc(s) with a fixation at the center of the screen for 250 ms. The locations of the discs were randomly selected from eight positions equidistant from a central fixation. Following a brief 900 ms blank retention interval, a test display with a color wheel appeared for the participant to respond. The participant needed to recall the color of a disc in the memory display at the location indicated by a cue and select the matching hue on the color wheel. After the participant responded, there was an 830 ms intertrial interval. The experiments included one practice trial and two formal blocks of 112 trials, where the set‐size and the probe context were counterbalanced and randomly intermixed. The probe context included whether the nontarget items were present or absent. In addition, a control experiment consisted of 56 trials was carried out to examine the performance of perceptual matching. On each trial of the control experiment, only one disc was presented and meanwhile the participant needed to select the matching hue on the color wheel. Based on the collected data, various indicators for VWM ability, such as the VWM capacity, the accuracy of reported hues, and the probability of retaining the cued item can be estimated by model fitting (Bays & Husain, 2008; van den Berg, Awh, & Ma, 2014; Zhang & Luck, 2008). All the experiments and estimation were done by Cam‐CAN (detailed information, Shafto et al., 2014). Since the putative VWM capacity for color is about four items (Alvarez & Cavanagh, 2004; Luck & Vogel, 1997, 2013), the capacity measure that obtained on four colored discs is most likely to capture individual differences among participants, and therefore here we adopted this measure for analyses. Additionally, the experimental paradigm was widely used for testing the performance on VWM (Mitchell & Cusack, 2018; Shafto et al., 2014).

### 2.3 Data preprocessing

1. R‐fMRI data: Unless stated otherwise, all functional imaging data preprocessing was performed using Statistical Parametric Mapping (SPM12, http://www.fil.ion.ucl.ac.uk/spm/), Resting‐State fMRI Data Analysis Toolkit (REST, http://rest.restfmri.net) (Song et al., 2011), and Data Processing Assistant for Resting‐State fMRI (Yan & Zang, 2010). The first six functional images were discarded to allow the signal stability (four participants were excluded for lacking volume number). The remaining R‐fMRI images were preprocessed as follows: (a) slice‐timing, (b) realignment (69 participants were excluded because of large head movement: >2 mm/2°), (c) spatial normalization to the Montreal Neurological Institute (MNI) space using the normalization parameters estimated in T1 segmentation (one participant was excluded for normalization failure caused by huge ventricle), (d) smoothing with a 4 mm full width at half maximum (FWHM) Gaussian kernel, (e) removing the linear trends, and (f) covariates regression (including Friston 24‐head motion parameters (Worsley et al., 1996), cerebrospinal fluid, white matter and global signals). After preprocessing, the ALFF was calculated for each participant (Zang et al., 2007). Briefly, we first converted the time series of every voxel to the frequency domain using a fast Fourier transformation. Then the square root of the power spectrum was computed and then averaged across 0.01–0.1 Hz. Thus, for each participant, there was an ALFF map.
2. T1‐weighted data: T1‐weighted MPRAGE data preprocessing included: (a) coregistration to the individual average functional image, (b) segmentation into gray matter, white matter, and cerebrospinal fluid, (c) modulating the resulting normalized gray matter images with Jacobian determinants of the deformation parameters, (d) smoothing GMV image with an 8 mm FWHM Gaussian kernel, and (e) resampling to 3 mm isotropic voxels to match the same voxel size as preprocessed R‐fMRI data. Thus, for each participant, there was a GMV map.
3. DWI data: DWI data were preprocessed using FSL (https://fsl.fmrib.ox.ac.uk/fsl/fslwiki) with the standard preprocessing procedure (Smith et al., 2004). The procedure included: (a) skull‐stripping, (b) simple‐motion and eddy‐current correction, (c) diffusion tensor/parameter calculation, and (d) spatial normalization. FA maps in the MNI space were generated for each individual. Eight participants were excluded for image artifacts, four of which overlapped with the participants whose R‐fMRI showed large head movement.

After removing unqualified data in preprocessing (i.e., large head movement or image artifacts), there were 547 participants (male = 269, age = 18–88) left for subsequent analyses. The demographics and behavioral data of the participants are summarized in Table 1.

**TABLE 1:** Demographics and behavioral performance of the participants

	Cam‐CAN Stage 2 dataset (*N* = 547)^a^ ^,^ ^b^
Gender (M/F)	269/278
Age (years)	53.95 ± 18.30 (18–88)
Visual working memory	2.51 ± 0.82 (0.00–4.00)

Abbreviation: Cam‐CAN, Cambridge Centre for Ageing and Neuroscience.

^a^ Behavioral and age data are presented as mean ± *SD* (minimum‐maximum).

^b^ 78 participants were excluded because of large head motion (*N* = 69), missing volumes (*N* = 4), huge ventricle (*N* = 1), and image artifacts in DWI (*N* = 8, 4 of which overlapped with the participants who had large head motion).

### 2.4 Model construction and feature analyses

**FIGURE 1:**
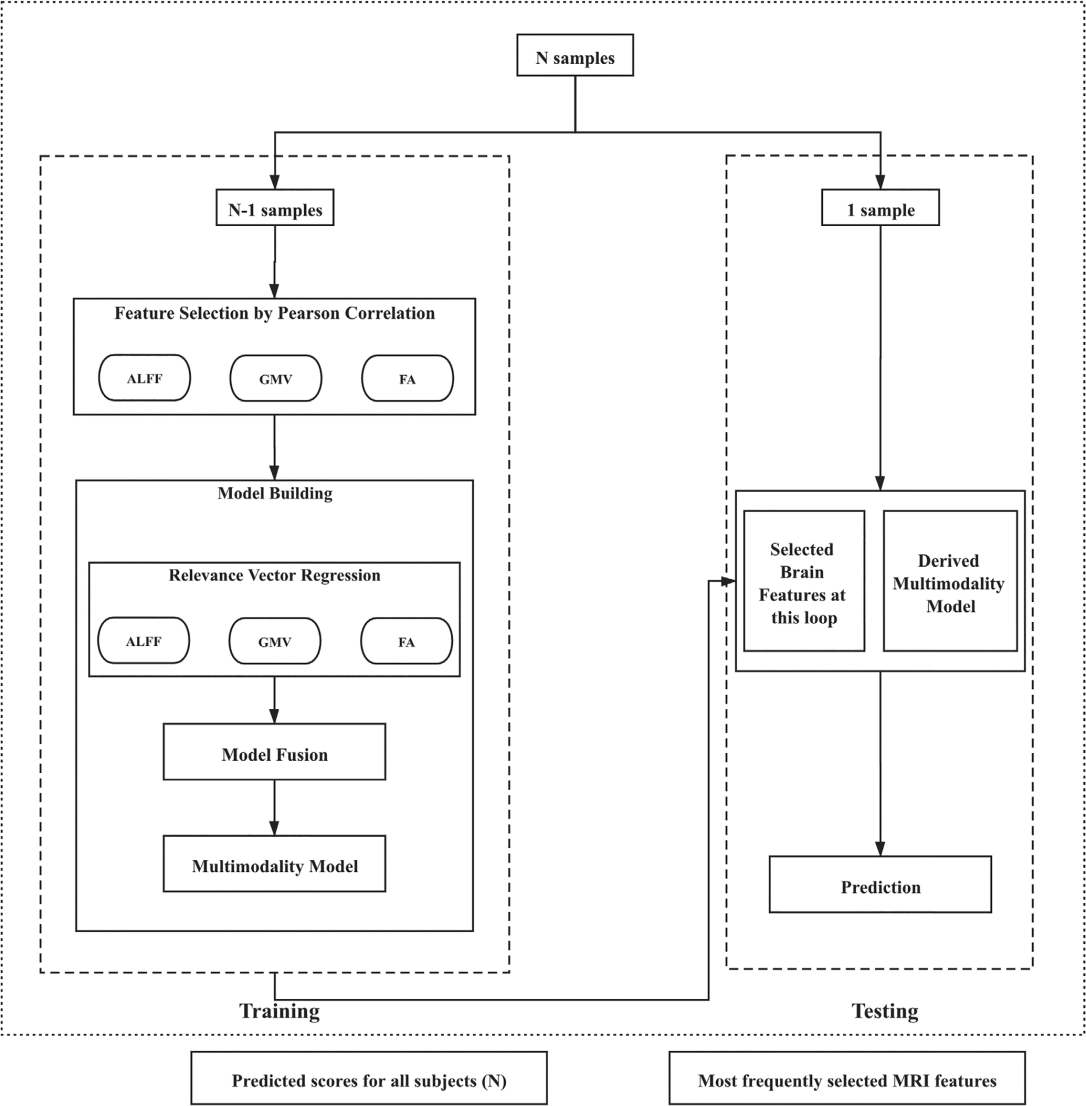
Model construction procedure. The step of model fusion was opted out in the case of building mono‐modality models, and the models to be fused were dependent on the modalities in use (i.e., ALFF + GMV, ALFF + FA, GMV + FA, or ALFF + GMV + FA). ALFF, amplitude of low‐frequency fluctuations; FA, fractional anisotropy; GMV, gray matter volume

The overall procedure of model construction is illustrated in Figure 1. First, we extracted 253,444 features from the multimodality voxel‐wise imaging data of each participant. Specifically, we first obtained the gray matter mask (*N*_voxels_ = 57,806) generated by a threshold of 0.2 on a priori gray matter probability map in SPM and the white matter mask (*N*_voxels_ = 137,832) generated by a predefined FA skeleton mask in FSL (FMRIB58_FA‐skeleton_1mm.nii). Then, for each participant, the ALFF values were extracted from the ALFF map and the GMV values were extracted from the GMV map, both of which were constrained within the gray matter mask. The FA values were extracted from the FA map, which was constrained within the FA skeleton mask. Hence, for each participant, there were 57,806 ALFF features; 57,806 GMV features; and 137,832 FA features. Second, the RVR method (Tipping, 2001) and the leave‐one‐out cross‐validation (LOOCV) technique (Pereira, Mitchell, & Botvinick, 2009) were employed to build prediction models of VWM based on the above features. We also assessed the effects of different cross‐validation regimes on the main results. Please refer to the “Validation Analyses” for details. For each fold of LOOCV, one participant was withheld to test the model trained on the other 546 participants. There were 547 folds in total and each participant was used as the test set in one fold. During the training session of the *i*th fold in LOOCV, we first normalized the regional features of the training sample (i.e., 546 participants) by using the z‐score approach on each modality separately. Then the Pearson correlations of the normalized features with the VWM scores were computed within the training sample. For each modality, the features with correlation significance beyond a corrected threshold (i.e., *p* = .05/number of features in the modality) were selected. Using the selected features in one modality, the RVR method was applied to train a mono‐modality model that predicted the VWM score. Three mono‐modality models, namely the ALFF, GMV, and FA models, were thus obtained. We then built four multimodality models by bagging the three mono‐modality models into three bimodality models (i.e., ALFF + GMV, ALFF + FA, GMV + FA) and one tri‐modality model (i.e., ALFF + GMV + FA). Using the bagging strategy of model averaging (Claeskens & Hjort, 2008; Hansen, 1999), the VWM scores predicted by each of these multimodality models were derived by averaging the VWM scores estimated by its composing mono‐modality models. This way, the bagging process allowed information from multiple modalities to integrate but did not interfere with the feature selection and training processes in each mono‐modality model, which reduced model uncertainty in the mono‐modality scenario and improved the prediction performance in both terms of accuracy and robustness. In the testing session of the *i*th fold in LOOCV, the features of the test set (i.e., data of the *i*th participant, *i* = 1, 2, …, 547) were normalized by the z‐score approach using means and standard deviations acquired from the training set, as suggested in previous studies (Cui & Gong, 2018). The above seven prediction models were applied to the normalized test set to predict the VWM score of the *i*th participant.

Finally, for each of the seven models, we evaluated the goodness of fit by calculating the correlation and the mean absolute error (MAE) between the observed and the predicted VWM scores of all the 547 participants. Specifically, the MAE was calculated as the mean of absolute differences between the observed and the predicted VWM scores. To validate the significance of the correlation, a permutation test (*N* = 1,000) was performed. In each permutation, the above modeling procedure was repeated with the association between the features and the observed VWM scores randomized (Dosenbach et al., 2010). Based on the null distribution built by the correlations obtained from the 1,000 permutations, the *p* value was estimated as the proportion of permutations that obtained a higher correlation than the tested model. Given that the correlation between the observed values and the predicted values is used as a measure of the model fit, model comparison can then be conducted by testing whether the correlations from two models are significantly different. Because the correlations of interest were measured on the same sample, the procedure of testing the difference between two dependent correlations described in Steiger (1980) was used. One‐sided *t* test based on equation (7) in Steiger (1980) was performed, given corresponding T_2_ statistics and *p* values. Based on the results of the above Steiger's method, an ultimate prediction model could be selected. To show the effectiveness and superiority of this ultimate model, we compared the prediction performance of the ultimate model with those of two other models obtained by using different modeling approaches but the same feature set, bagging strategy, and cross‐validation framework. The first model was built by linear regression (LR), which is a simple yet effective machine learning technique and is widely used to build baseline model (Seber & Lee, 2012). The second model was built by support vector regression (SVR), which is found to be one of the most promising machine learning techniques in a wide range of prediction tasks (Cui & Gong, 2018; Drucker, Surges, Kaufman, Smola, & Vapnik, 1997; Sui, Jiang, Bustillo, & Calhoun, 2020) and is the most similar RVR model homolog (Tipping, 2001).

The feature analyses aimed at identifying the brain regions that can effectively predict individual VWM capacity. To do this, the discriminative weight for each feature involved in the ultimate prediction model was estimated as its average weight across all folds in LOOCV (Dai et al., 2012; Dosenbach et al., 2010; Zeng et al., 2012). The effect of a voxel in relation to individual VWM capacity could then be quantified by the signs and the absolute values of the weights of the corresponding features. For better visualization of the results, we mapped voxel‐wise data onto a priori parcellation map for each modality. Specifically, ALFF and GMV results were mapped onto a 268‐node network (Shen, Tokoglu, Papademetris, & Constable, 2013) and the 268 nodes were further assigned to eight modules (Finn et al., 2015). FA results were mapped onto JHU‐ICBM DTI atlas (Mori, Wakana, Nagae‐Poetscher, & van Zijl, 2005). For each regional feature obtained, its discriminative weight was estimated as the mean discriminative weight of the features of the voxels involved in the corresponding node.

All the above analyses were implemented with the Pattern Recognition for Neuroimaging Toolbox (http://www.mlnl.cs.ucl.ac.uk/pronto/), the SPM12 toolbox (http://www.fil.ion.ucl.ac.uk/spm/), and our in‐house MATLAB code.

### 2.5 Validation analyses

To validate our main findings, we reconducted the main analyses on the same dataset using different cross‐validation regimes and preprocessing strategies. In terms of cross‐validation regimes, repeated 5‐fold and 10‐fold cross‐validation frameworks (*N* = 100) were used to avoid potential overoptimistic bias induced by LOOCV. In terms of preprocessing strategies, we considered the following three aspects. (a) Global signal regression. Previous studies have suggested that whether to remove the global signal or not might significantly alter the research results (Ibinson et al., 2015; Murphy & Fox, 2017; Power et al., 2014). We thus examined the results without removing the global signal. (b) Head motion exclusion. To avoid possible bias caused by different head motion exclusion criteria, we performed a spike‐regression‐based scrubbing in the original nuisance regression procedure during data preprocessing (Power, Schlaggar, & Petersen, 2015; Yan et al., 2013) with the criteria of a frame‐wise displacement (Jenkinson, Bannister, Brady, & Smith, 2002) above 0.5 mm. (c) Standard tract‐based spatial statistics (TBSS). Previous studies have suggested that TBSS could improve intersubject FA image alignment, making it more sensitive and interpretable for multisubject DTI analyses (Smith et al., 2006). Thus, we examined the results with TBSS performed before FA extraction. Using the normalized FA maps for each individual from the aforementioned DWI preprocessing method, we created a mean group‐wise FA image and projected the individual diffusion metric data onto it.

To further validate that our main findings were specific to VWM, we applied the machine learning pipeline proposed in the main study to predict two other cognitive functions that Cam‐CAN study measured (Shafto et al., 2014). One cognitive function was emotion regulation (ER). It was chosen because it is associated with executive functions as VWM does (Brown, Brockmole, Gow, & Deary, 2012; Bull & Scerif, 2001). The ER task required the participants to watch positive, neutral, and negative film clips and rate their emotional feelings afterward at both negative and positive scales of 0–100. Then the participants were asked to reappraise the negative film clips through reinterpreting their meaning in an emotional impact reducing way. Since reappraisal provided a measure of how well people regulate their negative emotions, an ER score can be calculated by comparing the ratings for the negative film clips at the watch and reappraise conditions. The other cognitive function used for validation was FI, which was selected due to the importance of working memory in it (Heitz, Unsworth, & Engle, 2005; Jaeggi, Buschkuehl, Jonides, & Perrig, 2008) and served as the baseline for comparison. The FI of each participant was measured by a standard form: the Cattell Culture Fair, Scale 2 Form A (Cattell & Cattell, 1973), containing four subtests on series completion, classification, matrices, and conditions, resulting in a score ranging from 0 to 46. More detailed descriptions of the tasks could be found in the previous study (Shafto et al., 2014). After applying the proposed pipeline to build prediction models for ER and FI, respectively, we compared the prediction performance and the distributions of discriminative features with those of the ultimate VWM model. Notably, in the model construction procedure for ER, the feature selection criterion was relaxed to *p* < .001 to ensure that at least 10 voxel‐wise features can be selected into the model from each modality.

## 3 RESULTS

**FIGURE 2:**
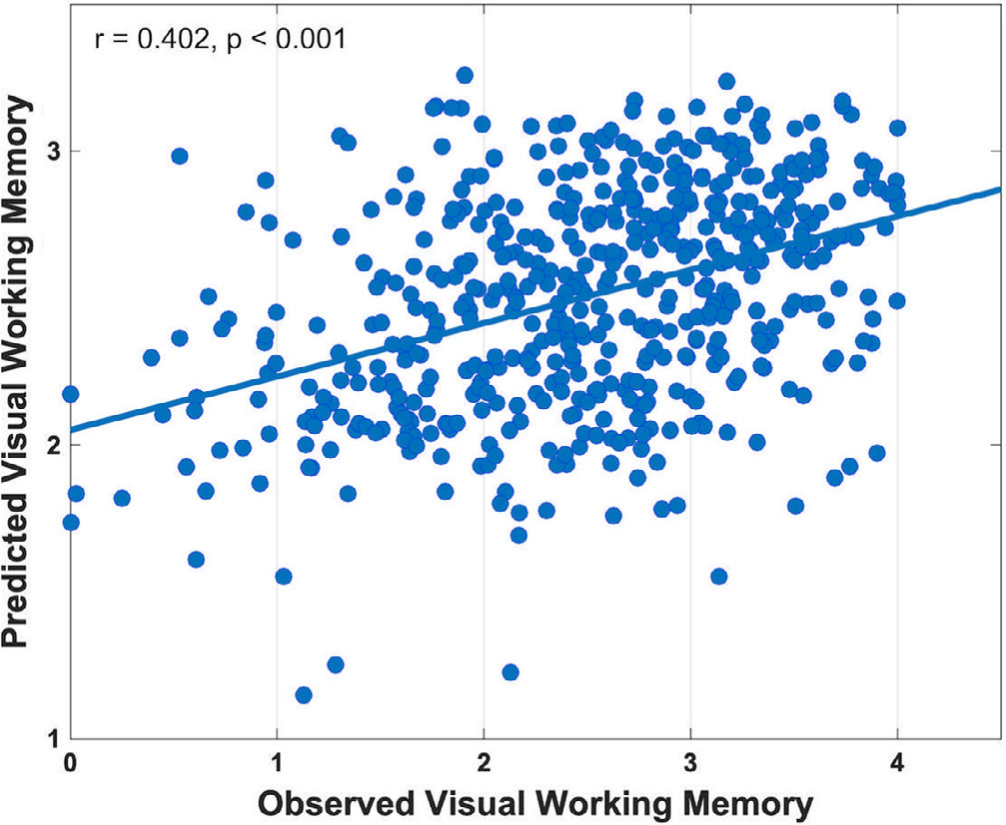
Correlation between the observed visual working memory (VWM) score and the VWM score predicted by the bagging model of three modalities. Related validation results were included in Supplementary Figures 1a–i

Table 2 tabulates the goodness of fit and the average number of features used by the seven prediction models. As can be seen, the multimodality models generally outperformed the mono‐modality models. In particular, the bimodality model GMV + FA (*r* = .414, *p* < .001; MAE = 0.599), the tri‐modality model ALFF + GMV + FA (*r* = .402, *p* < .001; MAE = 0.604), and the mono‐modality model GMV (*r* = .400, *p* < .001; MAE = 0.610) achieved the best predictive power, while the other models achieved prediction performance with the empirical‐predicted correlation r∈[0.297, 0.381] (*p* < .001) and MAE∈[0.613, 0.642]. The Steiger's method (Steiger, 1980) found that the prediction performance of the tri‐modality model had no significant difference with the models of GMV + FA and GMV (*p* > .1), but was significantly better than the other four models (*p* < .05). Further, when tested in the repeated 5‐ and 10‐fold cross‐validation frameworks (*N* = 100), the tri‐modality model achieved the highest predictive power with the smallest variance across repetitions (Supplementary Tables 1a and 1b; Supplementary Figures 1a and 1b). Taken together the above results and our research goal to identify VWM relevant neural basis from three different modalities, we selected the tri‐modality model as the ultimate prediction model for further analyses. A detailed plot of the observed VWM scores and the VWM scores predicted by this model is shown in Figure 2. To show the effectiveness and superiority of the above tri‐modality model, we built two additional tri‐modality models using the LR and SVR approaches. Note that except for replacing RVR with LR and SVR, respectively, the two additional models were trained and tested in exactly the same way as the tri‐modality RVR model. The Steiger's method reported that the performances of the tri‐modality LR model (*r* = .298, *p* < .001; MAE = 0.644; Supplementary Figure 1c) and the tri‐modality SVR model (*r* = .268, *p* < .001; MAE = 0.699; Supplementary Figure 1d) were significantly lower than that of the tri‐modality RVR model.

**TABLE 2:** Results of the seven prediction models

Modalities	*r*‐Value	Number of selected features			T_2_	*p*‐Value
		ALFF	GMV	FA		
ALFF	.297	118	—	—	5.170	1.64 × 10^−7^
GMV	.400	—	36,366	—	0.063	.475
FA	.362	—	—	19,918	2.029	.021
ALFF + GMV	.381	118	36,366	—	3.576	1.89×10^−4^
ALFF + FA	.367	118	—	19,918	1.918	.028
GMV + FA	.414	—	36,366	19,918	−1.167	.122
ALFF + GMV + FA	.402	118	36,366	19,918	—	—

*Note*: *r* value: Pearson correlation between the predicted and the observed visual working memory scores. Number of selected features: the average number of selected features across all the 547 folds in the cross‐validation procedure. T_2_: one‐sided two‐sample *t*‐test statistics based on Steiger's (1980) method, which was calculated by comparing the prediction performance of the corresponding model with the tri‐modality model. *p* value: significant level of the corresponding T_2_ statistic. Related validation results were included in Supplementary Tables 1 and 5.

Abbreviations: ALFF, amplitude of low‐frequency fluctuations; FA, fractional anisotropy; GMV, gray matter volume.

**FIGURE 3:**
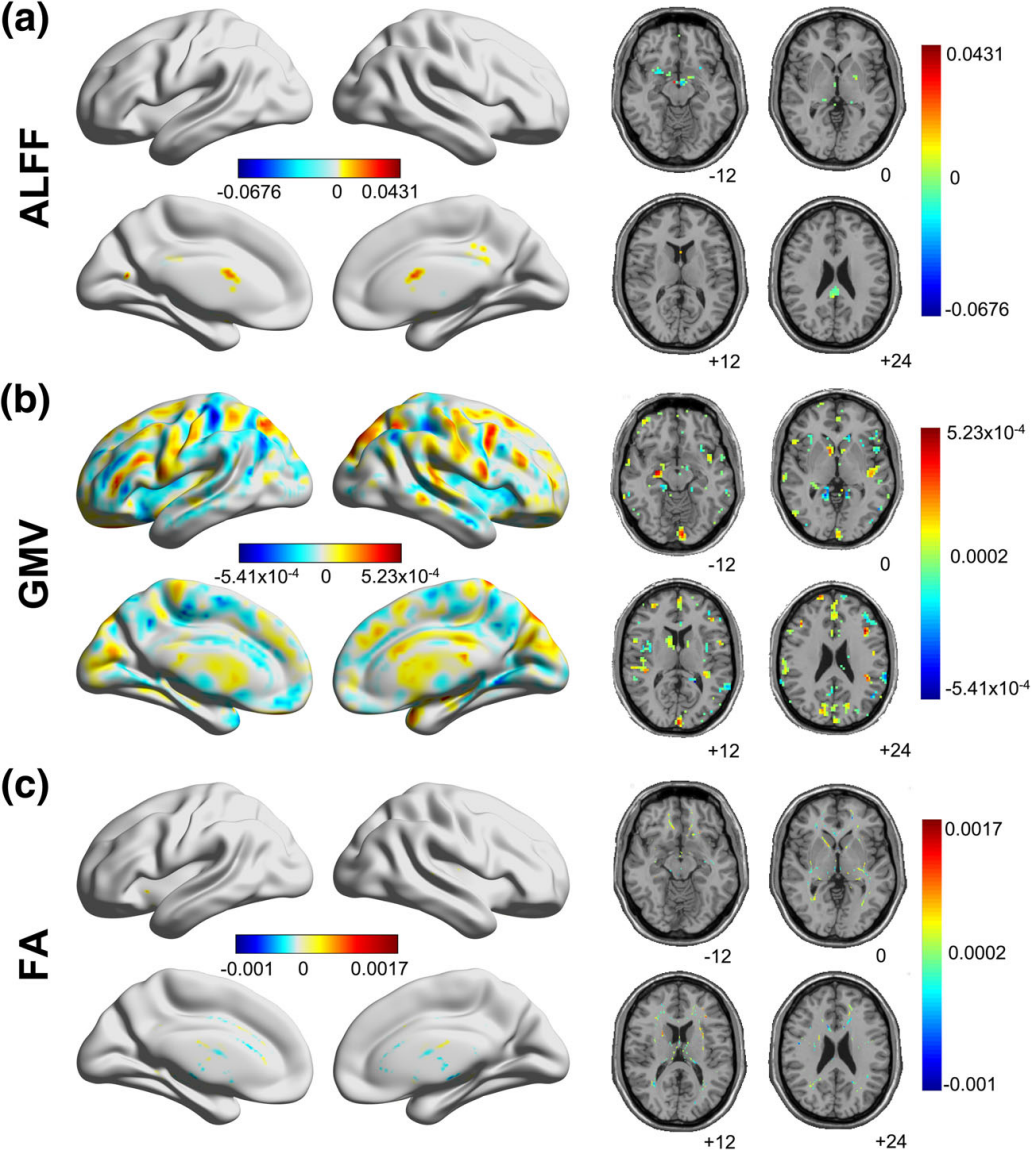
Discriminative weights of voxel‐wise features in (a) amplitude of low‐frequency fluctuations (ALFF), (b) gray matter volume (GMV), and (c) fractional anisotropy (FA). In all the three maps, warm color indicates positive weights, while cold color indicates negative weights. Darker color indicates larger absolute values of the weights. Related validation results were included in Supplementary Figures 2a–g

**FIGURE 4:**
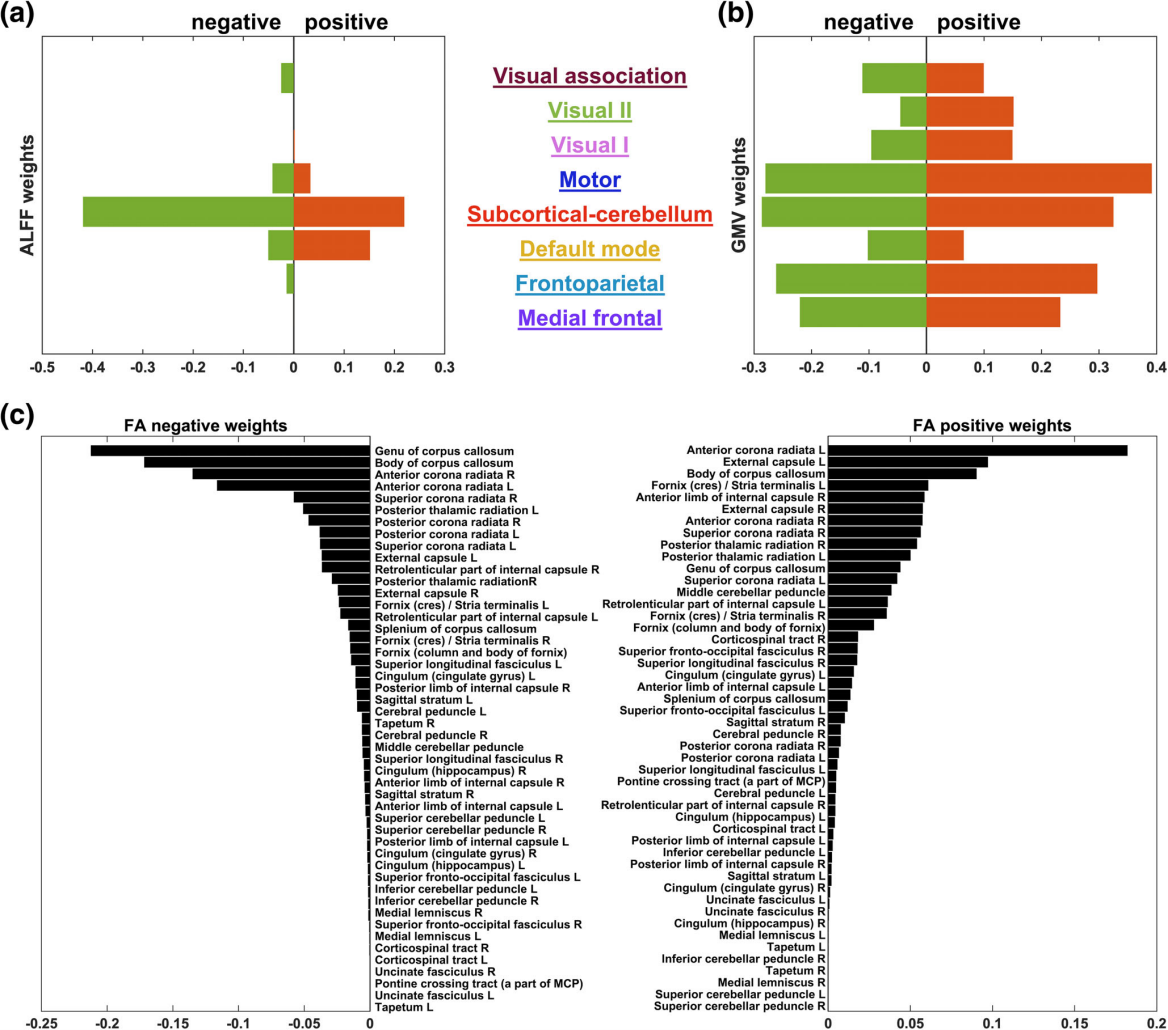
Respective sum of positively and negatively contributive weights from the (a) eight networks of amplitude of low‐frequency fluctuations (ALFF), (b) eight networks of gray matter volume (GMV), and (c) 48 fiber tracts of fractional anisotropy (FA). The network/fiber information was derived from the 268‐node functional atlas (Finn et al., 2015; Shen et al., 2013) and JHU‐ICBM DTI atlas (Mori et al., 2005), respectively. Related validation results were shown in Supplementary Figures 3a–g

In the ultimate prediction model, 118 ALFF features (*p* < .05/57,806); 36,366 GMV features (*p* < .05/57,806); and 19,918 FA features (*p* < .05/13,7832) were used. Figure 3 plots the weight distribution of these voxel‐wise features. For ALFF, the most contributive regions appeared in caudate, cerebellum, hippocampus, and cingulate gyrus. For GMV, the most contributive regions were in precuneus, postcentral gyrus, and middle frontal gyrus. For FA, the most contributive regions were from anterior corona radiata, corpus callosum, and external capsule. The voxel‐wise weights were then clustered into module/white matter tract weights according to the corresponding atlas in each modality for better visualization and explanation, as shown in Figure 4, with details of the top 10 most contributive regions tabulated in Tables 3, 4, 5. For ALFF, the most contributive (either positively or negatively, unless stated otherwise) regions were in the subcortical‐cerebellum, default mode and motor networks. For GMV, the most contributive regions were in the subcortical‐cerebellum, motor, frontoparietal, and medial frontal networks. For FA, the corpus callosum, anterior corona radiata, and external capsule made the greatest contributions.

**TABLE 3:** Top 10 contributive ALFF regions and their discriminative weights

Rank	Coordinate			Weight	Region
	x	y	z		
*Positive weight*						
1	−3	−84	45	0.0431	Near left cuneus
2	−15	−63	15	0.0364	Left calcarine
3	0	6	18	0.0318	Near left caudate
4	0	9	15	0.0308	Near left caudate
5	6	−6	−12	0.0289	Near right hippocampus
6	3	9	15	0.0251	Near right caudate
7	3	−42	27	0.0240	Right cingulate gyrus, posterior part
8	−15	−6	−9	0.0238	Near left hippocampus
9	0	6	15	0.0233	Near left caudate
10	0	3	18	0.0199	Near left caudate
*Negative weight*						
1	−42	−75	−21	−0.0676	Left cerebellum Crus1
2	−48	−36	−30	−0.0411	Near left cerebellum Crus1
3	12	−24	−39	−0.0367	Near right cerebellum 10
4	−18	−15	−18	−0.0349	Left hippocampus
5	12	−54	−60	−0.0311	Near right cerebellum 9
6	9	−24	−42	−0.0311	Near right cerebellum 10
7	−6	−6	−12	−0.0267	Near left hippocampus
8	−3	−9	−12	−0.0261	Near left hippocampus
9	3	−12	3	−0.0240	Right thalamus
10	−27	12	−12	−0.0210	Right insula

*Note*: Related validation results were included in Supplementary Tables 2 and 6.

Abbreviation: ALFF, amplitude of low‐frequency fluctuations.

**TABLE 4:** Top 10 contributive GMV regions and their discriminative weights

Rank	Coordinate			Weight	Region
	x	y	z		
*Positive weight*					
1	0	−39	60	0.000523	Left precuneus
2	39	9	42	0.000522	Right middle frontal gyrus
3	36	12	45	0.000503	Right middle frontal gyrus
4	0	−36	60	0.000501	Right paracentral lobule
5	−33	−21	39	0.000493	Near left postcentral gyrus
6	−39	21	24	0.000491	Left inferior frontal gyrus (triangular)
7	0	−39	63	0.000485	Near left Precuneus
8	−15	45	45	0.000483	Left superior frontal gyrus
9	−15	45	42	0.000479	Left superior frontal gyrus
10	27	−12	−12	0.000478	Right hippocampus
*Negative weights*					
1	−39	−30	54	−0.000541	Left postcentral gyrus
2	−21	−45	12	−0.000507	Near left precuneus
3	15	−15	6	−0.000499	Right thalamus
4	−36	−30	54	−0.000497	Left postcentral gyrus
5	−39	−30	51	−0.000477	Left postcentral gyrus
6	−39	−30	57	−0.000462	Left postcentral gyrus
7	−12	−15	3	−0.000459	Left thalamus
8	−36	−30	57	−0.000459	Left postcentral gyrus
9	−12	−18	6	−0.000458	Left thalamus
10	−51	−60	18	−0.000453	Left middle temporal gyrus

*Note*: Related validation results were included in Supplementary Tables 3 and 7.

Abbreviation: GMV, gray matter volume.

**TABLE 5:** Top 10 contributive FA regions and their discriminative weights

Rank	Coordinate			Weight	Region
	x	y	z		
*Positive weight*					
1	36	42	−5	0.00170	Near right anterior corona radiata
2	35	43	−4	0.00167	Near right anterior corona radiata
3	36	43	−5	0.00162	Near right anterior corona radiata
4	35	42	−4	0.00159	Near right anterior corona radiata
5	36	42	−4	0.00156	Near right anterior corona radiata
6	35	43	−3	0.00156	Near right anterior corona radiata
7	35	42	−3	0.00154	Near right anterior corona radiata
8	34	43	−3	0.00149	Near right anterior corona radiata
9	−24	24	−13	0.00143	Near left external capsule
10	36	43	−4	0.00143	Near right anterior corona radiata
*Negative weight*					
1	11	30	−4	−0.00104	Genu of corpus callosum
2	3	−19	−6	−0.00104	Near right cerebral peduncle
3	0	0	12	−0.00101	Fornix (column and body of fornix)
4	3	−19	−5	−0.00100	Near right cerebral peduncle
5	9	26	−5	−0.000959	Genu of corpus callosum
6	25	−52	29	−0.000955	Near right posterior corona radiata
7	3	−18	−5	−0.000946	Near right cerebral peduncle
8	−8	8	26	−0.000932	Body of corpus callosum
9	11	29	−6	−0.000929	Genu of corpus callosum
10	9	29	−3	−0.000922	Genu of corpus callosum

*Note*: Related validation results were included in Supplementary Tables 4 and 8.

Abbreviation: FA, fractional anisotropy.

We assessed the reproducibility of our main results under different cross‐validation regimes (repeated 5‐ and 10‐fold cross‐validation) and preprocessing strategies (without global signal removal, motion scrubbing, TBSS). Regarding the two different cross‐validation regimes, we found that the advantage of multimodality models against mono‐modality models maintained, with the tri‐modality model achieving the best performance in both cases (5‐fold: *r* = .404 ± 0.013, *p* < .001, MAE = 0.602 ± 0.005, Supplementary Table 1a; 10‐fold: *r* = .408 ± 0.010, *p* < .001, MAE = 0.601 ± 0.004, Supplementary Table 1b). As for the three preprocessing strategies (Supplementary Tables 1c–1e), like in the main analysis, the multimodality models GMV + FV and ALFF + GMV + FA had the highest predictive power (.402 ≤ *r* ≤ .414, *p* < .001, 0.599 ≤ MAE ≤0.605) given that the global signal was not removed or the TBSS strategy was used. It was only in the case of motion scrubbing could the mono‐modality model GMV (*r* = .400, *p* < .001, MAE = 0.608) slightly outperform the tri‐modality model (*r* = .364, *p* < .001, MAE = 0.611), while the bi‐modality model GMV + FV remained to be the best one (*r* = .414, *p* < .001, MAE = 0.599). In the aspect of feature analyses, with the repeated 5‐ and 10‐fold cross‐validation, the number of selected features for GMV and FA dropped to around 400 and 200, respectively (Table 2, Supplementary Tables 1a and 1b). Yet the number of voxels selected for each modality remained stable across the three preprocessing strategies, that is, around 100 for ALFF, around 36,000 for GMV, and around 19,000 for FA (Table 2, Supplementary Tables 1c–1e). We also found consistent spatial patterns of the contributive regions in each modality across the above five validation conditions (Figure 3, Supplementary Figures 2a–2e). For ALFF, the most contributive regions remained the same except for the case of motion scrubbing (Table 3, Supplementary Tables 2a–2d), in which the contributive regions shifted to cingulum gyrus, putamen and pallidum. For GMV, the most contributive regions were the same across the three different preprocessing strategies, but shifted to frontal gyrus and inferior occipital regions when the two cross‐validation regimes were used (Table 4, Supplementary Tables 3a and 3b). For FA, the most contributive regions remained for all validation conditions (Table 4, Supplementary Tables 4a–4c). Besides the spatial locations of contributive regions, our findings regarding the network‐level contributions also maintained under the five validation conditions (Figure 4, Supplementary Figures 3a–3e). Only the motion scrubbing process showed a relatively large influence on the pattern of positively contributive networks in ALFF. The default mode network instead of the subcortical‐cerebellum network became the largest positively contributor. In summary, the validation analyses showed that our main findings were robust and reproducible under different cross‐validation regimes and preprocessing strategies. For more details, please refer to Supplementary Materials.

We also assessed the specificity of our main findings through comparison with the findings obtained by using the proposed pipeline on ER and FI. As shown in Supplementary Table 5 and Supplementary Figures 1h and 1i, the correlation between the predicted and the actual ER scores was 0.085 (*p* = .099), and that between the predicted and the actual FI scores was 0.656 (*p* < .001). Most importantly, the discriminative features drawn from the prediction models for the three cognitive functions were totally different (Tables 3, 4, 5, Figures 3 and 4; Supplementary Tables 6–8, Supplementary Figures 2f and 2g, Supplementary Figures 3f and 3g), which further confirmed the exclusiveness of our findings. Specifically, different from the contributor pattern in the VWM model where high level cognitive networks showed higher contributions, in the model predicting ER, the ALFF and GMV features in the basic sensory‐motor and subcortical‐cerebellum networks were more contributive than those in the higher cognitive networks including frontoparietal and medial frontal networks. For ALFF, the most positively contributive regions were in visual, motor, subcortical‐cerebellum, and frontoparietal networks, and the most negatively contributive regions were in subcortical‐cerebellum, motor, and medial frontal networks. For GMV, the most contributive regions were in motor, subcortical‐cerebellum, and visual networks. For FA, the most contributive regions were in external capsule, internal capsule, and cingulate gyrus. For FI, the ALFF features in higher cognitive networks contributed more than they did for VWM. In particular, the subcortical‐cerebellum, default mode, and frontoparietal networks were the most contributive regions in ALFF for FI. For GMV, the most contributive regions were in subcortical‐cerebellum, motor, frontoparietal, and medial frontal networks. For FA, the most contributive regions were in corpus callosum, external capsule, and corona radiata.

## 4 DISCUSSION

By successfully building a prediction model on individual VWM capacity using multimodal MRI data and machine learning techniques, we demonstrated that specific individual cortical morphologies are associated with subject‐level variation in VWM. Key features identified for ALFF were in the subcortical‐cerebellum network, default mode network, and motor network. Key features identified for GMV were more widespread, with higher cognitive networks playing the main role. For FA, key features were mainly identified from white matter fibers of corpus callosum, anterior corona radiata, and external capsule. These results were highly replicable using different cross‐validation and preprocessing schemes. Although in scrubbing data, the three higher cognitive networks revealed more discriminant validity for ALFF features. These results revealed rich information from both structural and functional perspectives on individual differences in VWM capacity using task‐independent data, which may provide further implications on individual VWM and cognition research.

### 4.1 Model behaviors

In this article, we adopted the RVR model, embedded with correlation‐based feature selection and average bagging, to predict individual VWM capacity using multimodal imaging data. The multimodality models generally outperformed the mono‐modality models (Table 2 and Figure 2), suggesting that multimodal imaging data embraces richer information. This result also supports our hypothesis that multimodality offers more comprehensive idiosyncrasies in the neural basis of VWM. Note that the performance of the tri‐modality model was significantly better (*p* < .05) than all the other models except for the two involving the GMV features (i.e., GMV and GMV + FA). This phenomenon indicates that GMV is a prominent indicator for distinguishing individual VWM capacity, which is in line with the results of former studies (Dimond, Perry, Iaria, & Bray, 2019; Machizawa et al., 2020). It also provides support for previous findings that GMV atrophy could be a sensitive biomarker in memory related disorders such as Alzheimer's disease (Jack et al., 2013). Meanwhile, the insignificant difference between the two multimodality models (GMV + FA vs. ALFF + GMV + FA) suggests tight associations (e.g., collinearity) between the functional pattern (reflected by ALFF) and the structural pattern (reflected by GMV and FA) regarding VWM. This is expected because a wealth of research has shown that white matter microstructure links discreet brain areas and thus regulates brain functions (Gu et al., 2015; Tang & Bassett, 2018). In fact, with the predictive power of the multimodality models not equal to the sum of the power of the related mono‐modality models, we can also draw the above suggestion: the information provided by different modalities was, on the one hand, complement to each other, while on the other hand, overlapping to some extent. Similar results were also found in previous machine learning studies using imaging data (Dai et al., 2012; Ecker et al., 2010). Considering that the tri‐modality model performed the best in repeated 5‐ and 10‐fold cross‐validation (Supplementary Tables 1a and 1b; Supplementary Figures 1a and 1b) and one of the purposes of our study was to find the most predictive brain regions for individual VWM from three different modalities, the tri‐modality model was selected as the ultimate prediction model. The effectiveness of this tri‐modality model was further confirmed from three aspects: (a) robustness regarding three different preprocessing strategies (Supplementary Tables 1c–1e; Supplementary Figures 1e–1g); (b) superiority against the models built by LR (a baseline algorithm) and SVR (the kin counterpart of RVR) (Supplementary Figures 1c and 1d); and (c) specificity in contrast to prediction of ER and FI (Supplementary Table 5; Supplementary Figures 1h and 1i), both of which are cognitive indicators related to executive functions as VWM is.

### 4.2 Identified potential neuromarkers

Our results found that in both ALFF and GMV (Tables 3 and 4; Figures 3a,b and 4a,b), the most contributive features in the VWM prediction model were often associated with regions of the subcortical‐cerebellum network, which revealed the crucial function of subcortical‐cerebellum network in predicting individual VWM capacity. This is consistent with previous findings on the cognitive functions of cerebellum and subcortical regions. For example, Habas et al. (2009) reported that cerebellum was involved in various kinds of nonmotor functions, supported by interaction with basal ganglia and other brain regions (Bostan, Dum, & Strick, 2013). Sobczak‐Edmans et al. (2016) showed that different parts of the cerebellum contributed to different VWM processes, including encoding and maintenance. Houk et al. (2007) found that subcortical loops through basal ganglia and cerebellum were required in encoding the sequence of visual objects into a serial order of working memory. Further, our results showed that subregions of subcortical‐cerebellum network were related to the VWM capacity in different ways (positively or negatively), suggesting that different parts of the subcortical‐cerebellum network might act differently when performing VWM‐related tasks. Regions in the default mode network were also found contributive to VWM prediction. Most of them were positively correlated with VWM capacity, while a few exhibited negative correlation. Such a finding indicates that to facilitate VWM performance, most parts of the default mode network are activated while some other parts are suppressed. Further, it suggests that different parts of the default mode network might interact with different brain regions and participate in different levels of the VWM process. This is consistent with the findings of previous studies, which reported significant correlation between VWM performance and coordination within the default mode network and between the default mode network and other networks (Greicius, Krasnow, Reiss, & Menon, 2003; Sambataro et al., 2010; Uddin, Kelly, Biswal, Castellanos, & Milham, 2009). It is also supportive of the findings that impairment in the default mode network was related to the VWM capacity reduction in brain disorders such as depression and schizophrenia (Whitfield‐Gabrieli & Ford, 2012). Furthermore, it is worth noting that frontoparietal network showed a negative contribution to VWM prediction. Studies have shown that the activity between the default mode and frontoparietal networks during working memory and other tasks tended to be anticorrelated (Murphy, Bertolero, Papadopoulos, Lydon‐Staley, & Bassett, 2020; Uddin et al., 2009). This confirms our finding that a large portion of the contribution from the default model network was positive and the contribution from the frontoparietal network was negative. The motor network also made a great contribution to VWM prediction. Previous studies demonstrated that motor network was responsible for cognition, and it participated in attention and execution through connections with other networks (Jeannerod, 2001; van den Heuvel & Hulshoff Pol, 2010). Following these findings, our results on the strong contribution of motor network to VWM prediction reflected the task action, during which participants responded by clicking on the right color on the panel.

Aligned with the findings in ALFF and GMV, the white matter tract found using FA may suggest the potential pathways that link different networks regarding VWM (Table 5; Figures 3c and 4c). Anterior corona radiata, the most positively contributive fiber, was shown to play an important role in encoding visual information during VWM tasks (Katshu et al., 2017). Moreover, right anterior corona radiata was found significantly correlated with gaze error variability when doing the task (Maruta, Suh, Niogi, Mukherjee, & Ghajar, 2010), which further implies its importance in VWM. Corpus callosum, the major negatively contributive fiber, was shown to be distinctively associated with VWM (Menegaux et al., 2017). Golestani et al. (2014) have also shown that corpus callosum underlay the integration of visual information across the right and left visual fields. External capsule was shown to be the second positively contributive fiber to VWM. Studies have shown that external capsule was significantly associated with working memory (Charlton, Barrick, Lawes, Markus, & Morris, 2010; Nolze‐Charron et al., 2020).

For the validation studies about cross‐validation regimes and preprocessing strategies, the most contributive networks maintained except for the case of motion scrubbing (Supplementary Figure 3d). In scrubbing data, positive contributions for all the networks increased. This might be due to the interpolation of scrubbing decreased the power of some voxels. Overall, this further validates the generalizability and robustness of our findings. For the validation analyses regarding model specificity, the networks and regions contributive to the model that successfully predicted FI showed different patterns compared with VWM (Supplementary Tables 6b, 7b, and 8b; Supplementary Figures 2g and 3g; Tables 3, 4, 5, Figure 4). We found that the contributive networks of ALFF and GMV to FI were different from those contributive to VWM. Specifically, the frontal parietal and medial frontal networks of ALFF were more contributive to FI compared with VWM, and this is in line with previous findings that FI was associated with frontal brain (Finn et al., 2015; Woolgar et al., 2015). Meanwhile, we also found overlapping contributive regions for predicting VWM and FI. This is not a surprise as Fukuda et al. (2010) reported that VWM accounted for 43% of individual differences in global FI, and other studies have also revealed the close association between FI and VWM tasks (Heitz et al., 2005; Jaeggi et al., 2008). The overlapping regions included the subcortical‐cerebellum network in ALFF and GMV, and external capsule, corona radiata, and corpus callosum in FA. Takagi, Hirayama, and Tanaka (2019) have shown the state‐unspecific property of the subcortical‐cerebellum network across different cognitive tasks, revealing a general association of the subcortical‐cerebellum network with cognition. Consistently, Avery et al. (2019) also found that the subcortical‐cerebellum network was a discriminative predictor for both working memory and FI. Previous studies suggested that FI is associated with corpus callosum integrity (Nikolaidis et al., 2017; Wolf et al., 2014) and could be improved via increasing corpus callosum integrity during working memory training (Aydin, Uysal, Yakut, Emiroglu, & Yilmaz, 2012; Peng, Mo, Huang, & Zhou, 2017; Takeuchi et al., 2010). It was also observed that corpus callosum integrity could predict FI through working memory (Privado et al., 2014). Studies have shown that corona radiata integrity was associated with FI (Colom, Karama, Jung, & Haier, 2010; Nikolaidis et al., 2017). Tang et al. (2010) also found that activated corona radiata during working memory task was highly correlated with FI. As for external capsule, it is considered associated with executive functions (Mayo et al., 2019; Nolze‐Charron et al., 2020), and both VWM and FI are known to be correlated with executive functions (Brown et al., 2012; Brydges, Reid, Fox, & Anderson, 2012; Garlick & Sejnowski, 2006). Therefore, the overlapping contributive regions we found for predicting VWM and FI were actually in good match with previous findings.

### 4.3 Methodological issues and future directions

We chose the RVR method with a linear kernel to establish the VWM prediction model from the imaging data with high dimension but a relatively small sample size. Although this linear model already obtained satisfying prediction performance and was straightforward to interpret, given a larger sample, future studies might consider using nonlinear models to better capture the relationship between neural activities and individual VWM capacity. Besides, the feature selection method and the bagging method used in this article were effective but naïve. For better prediction performance, more complicated techniques can be used in future studies. It is also worth noting that the proposed model was built solely based on the dataset from the Cam‐CAN study. Although the model was trained, evaluated, and validated following strict protocols, it remains an open issue whether the model can perform well on imaging data from other populations and other sites. Therefore, it would be important to perform further validation on an independent dataset in future studies to examine the robustness and generalization ability of the proposed model, which might greatly increase the impact of the current findings.

Due to the sensibility of ALFF, GMV, and FA in predicting individual cognitive abilities (Zang et al., 2007), this article extracted the voxel‐wise data of the three modalities at the whole‐brain level to predict individual VWM capacity. Our results showed that many of the most predictive regions located in the subcortical‐cerebellum network, default mode network, and motor network. Moreover, different regions in the same network could affect the VWM capacity in different ways. Based on these findings, an interesting future direction is to explore whether and how the connectivity patterns of these predictive regions determine their influence on the VWM capacity and how they interact when performing VWM‐related tasks.

## 5 CONCLUSION

In summary, we combined multimodal imaging data of healthy participants to investigate individual VWM capacity using a well‐designed machine learning pipeline that included feature selection, RVR, cross‐validation, and model fusion. Using voxel‐wise features extracted from the whole brain in three modalities, we can predict individual VWM capacity with correlation at 0.402 (*p* < .001). Moreover, we identified several key predictors to individual VWM capacity from both functional and structural perspectives, which can serve as potential neuromarkers of VWM and might provide further implications for future related research.

## CONFLICT OF INTEREST

The authors declare no conflict of interest.

## ETHICS STATEMENT

All study data were obtained from repositories of publicly available data. These data were originally acquired with written informed consent of all studied participants.

## Supporting information


**Appendix** **S1.** Supporting Information.

## Data Availability

All the data used in this study are collected from a published database Cambridge Centre for Ageing and Neuroscience (Cam‐CAN) research. The raw data are available at https://camcan-archive.mrc-cbu.cam.ac.uk/dataaccess/.
