# Supplementary material for: Predicting visual working memory with multimodal magnetic resonance imaging
Source: Hum Brain Mapp. 2020 Dec 5;42(5):1446–62. doi: 10.1002/hbm.25305 (PMC7927291; doi:10.1002/hbm.25305)
Supplement: Supplementary file 1 — Appendix S1. Supporting Information. [file HBM-42-1446-s001.docx]

**STable 1a. Results of the seven prediction models on 5-fold cross-validation**

| **Modalities** | **r value** | **Number of selected features** | | | **T_2_** | **P value** |
| --- | --- | --- | --- | --- | --- | --- |
|  |  | **ALFF** | **GMV** | **FA** |  |  |
| **ALFF** | **0.334±0.023**  (0.282 - 0.389) | 61 | - | - | 2.775 | 0.003 |
| **GMV** | **0.372±0.028** (0.262 - 0.433) | - | 380 | - | 1.083 | 0.140 |
| **FA** | **0.332±0.017** (0.278 - 0.365) | - | - | 189 | 3.630 | 1.50×10^-4^ |
| **ALFF+GMV** | **0.393±0.017** (0.347 - 0.4289) | 61 | 380 | - | 0.397 | 0.346 |
| **ALFF+FA** | **0.377±0.015** (0.339 - 0.413) | 61 | - | 189 | 2.560 | 0.005 |
| **GMV+FA** | **0.389±0.016** (0.349 - 0.426) | - | 380 | 189 | 1.268 | 0.103 |
| **ALFF+GMV+FA** | **0.404±0.013** (0.371 - 0.431) | 61 | 380 | 189 | - | - |

r value: Pearson correlation between the predicted and the observed visual working memory scores. It is presented as mean ± SD (minimum-maximum) of 100 times iterations; number of selected features: the average number of selected features across all the 100 iterations of 5 folds in the cross-validation procedure

**STable 1b. Results of the seven prediction models on 10-fold cross-validation**

| **Modalities** | **r value** | **Number of selected features** | | | **T_2_** | **P value** |
| --- | --- | --- | --- | --- | --- | --- |
|  |  | **ALFF** | **GMV** | **FA** |  |  |
| **ALFF** | **0.342±0.015** (0.30377 - 0.374) | 87 | - | - | 2.890 | 0.002 |
| **GMV** | **0.372±0.022** (0.3186 - 0.416) | - | 429 | - | 1.299 | 0.097 |
| **FA** | **0.342±0.010** (0.31107 - 0.363) | - | - | 247 | 3.323 | 4.756×10^-4^ |
| **ALFF+GMV** | **0.395±0.014** (0.36431 - 0.427) | 87 | 429 | - | 0.733 | 0.232 |
| **ALFF+FA** | **0.385±0.010** (0.35755 - 0.403) | 87 | - | 247 | 2.343 | 9.749×10^-3^ |
| **GMV+FA** | **0.394±0.012** (0.36221 - 0.416) | - | 429 | 247 | 1.130 | 0.219 |
| **ALFF+GMV+FA** | **0.408±0.010** (0.38506 - 0.425) | 87. | 429 | 247 | - | - |

r value: Pearson correlation between the predicted and the observed visual working memory scores. It is presented as mean ± SD (minimum-maximum) of 100 times iterations; number of selected features: the average number of selected features across all the 100 iterations of 10 folds in the cross-validation procedure

**STable 1c. Results of the seven prediction models on global signal not removed data**

| **Modalities** | **r value** | **Number of selected features** | | | **T_2_** | **P value** |
| --- | --- | --- | --- | --- | --- | --- |
|  |  | **ALFF** | **GMV** | **FA** |  |  |
| **ALFF** | 0.306 | 113 | - | - | 4.832 | 8.812×10^-7^ |
| **GMV** | 0.400 | - | 36366 | - | 0.314 | 0.377 |
| **FA** | 0.362 | - | - | 19918 | 2.285 | 0.011 |
| **ALFF+GMV** | 0.387 | 113 | 36366 | - | 3.360 | 4.171×10^-4^ |
| **ALFF+FA** | 0.373 | 113 | - | 19918 | 1.703 | 0.045 |
| **GMV+FA** | 0.414 | - | 36366 | 19918 | -0.776 | 0.219 |
| **ALFF+GMV+FA** | 0.406 | 113 | 36366 | 19918 | - | - |

r value: Pearson correlation between the predicted and the observed visual working memory scores; number of selected features: the average number of selected features across all the 547 folds in the cross-validation procedure

**STable 1d. Results of the seven prediction models on scrubbing data**

| **Modalities** | **r value** | **Number of selected features** | | | **T_2_** | **P value** |
| --- | --- | --- | --- | --- | --- | --- |
|  |  | **ALFF** | **GMV** | **FA** |  |  |
| **ALFF** | 0.087 | 186 | - | - | 8.874 | <10^-10^ |
| **GMV** | 0.400 | - | 36366 | - | -1.891 | 0.030 |
| **FA** | 0.362 | - | - | 19918 | 0.123 | 0.451 |
| **ALFF+GMV** | 0.311 | 186 | 36366 | - | 5.664 | 1.198×10^-7^ |
| **ALFF+FA** | 0.295 | 186 | - | 19918 | 3.902 | 5.374×10^-5^ |
| **GMV+FA** | 0.414 | - | 36366 | 19918 | 4.017 | 3.361×10^-5^ |
| **ALFF+GMV+FA** | 0.365 | 186 | 36366 | 19918 | - | - |

r value: Pearson correlation between the predicted and the observed visual working memory scores; number of selected features: the average number of selected features across all the 547 folds in the cross-validation procedure

**STable 1e. Results of the seven prediction models on TBSS**

| **Modalities** | **r value** | **Number of selected features** | | | **T_2_** | **P value** |
| --- | --- | --- | --- | --- | --- | --- |
|  |  | **ALFF** | **GMV** | **FA** |  |  |
| **ALFF** | 0.297 | 118 | - | - | 5.014 | 3.602×10^-7^ |
| **GMV** | 0.400 | - | 36366 | - | 0.066 | 0.474 |
| **FA** | 0.355 | - | - | 19608 | 2.284 | 0.011 |
| **ALFF+GMV** | 0.381 | 118 | 36366 | - | 3.552 | 2.080×10^-4^ |
| **ALFF+FA** | 0.368 | 118 | - | 19608 | 1.836 | 0.033 |
| **GMV+FA** | 0.411 | - | 36366 | 19608 | 0.891 | 0.187 |
| **ALFF+GMV+FA** | 0.402 | 118 | 36366 | 19608 | - | - |

r value: Pearson correlation between the predicted and the observed visual working memory scores; number of selected features: the average number of selected features across all the 547 folds in the cross-validation procedure

**STable 2a. Top 10 contributive ALFF regions and their discriminative weights on 5-fold cross-validation**

| **Rank** | **MNI Coordinate** | | | **Weight** | **Region** |
| --- | --- | --- | --- | --- | --- |
|  | **x** | **y** | **z** |  |  |
| *Positive weight* | | | | | |
| **1** | 0 | 6 | 15 | 0.0402 | near left caudate |
| **2** | 0 | 9 | 15 | 0.0377 | near left caudate |
| **3** | 0 | 6 | 18 | 0.0282 | near left caudate |
| **4** | 0 | 3 | 18 | 0.0162 | near left caudate |
| **5** | 3 | -39 | 27 | 0.0144 | Right posterior cingulate gyrus |
| **6** | 3 | -42 | 27 | 0.0120 | Right posterior cingulate gyrus |
| **7** | -3 | 9 | 6 | 0.00979 | near left caudate |
| **8** | -24 | -6 | 3 | 0.00767 | Left Pallidum |
| **9** | 0 | -27 | 30 | 0.00763 | near left median cingulate |
| *Negative weight* | | | | | |
| **10** | 9 | -24 | -39 | -0.0375 | near right cerebelum 10 |
| **1** | -42 | -75 | -21 | -0.0332 | Left cerebelum Crus1 |
| **2** | 12 | -24 | -39 | -0.0323 | near right cerebelum 10 |
| **3** | -18 | -15 | -18 | -0.0281 | Left hippocampus |
| **4** | 24 | 9 | -12 | -0.0260 | Right olfactory |
| **5** | 12 | -27 | -39 | -0.0203 | near right cerebelum 10 |
| **6** | 6 | -24 | -42 | -0.0186 | near right Cerebelum 9 |
| **7** | 27 | 9 | -12 | -0.0148 | Right olfactory |
| **8** | 9 | -24 | -42 | -0.0130 | near right cerebelum 10 |
| **9** | -3 | -6 | -12 | -0.0122 | near left hippocampus |
| **10** | 9 | -24 | -39 | -0.0375 | near right cerebelum 10 |

**STable 2b. Top 10 contributive ALFF regions and their discriminative weights on 10-fold cross-validation**

| **Rank** | **MNI Coordinate** | | | **Weight** | **Region** |
| --- | --- | --- | --- | --- | --- |
|  | **x** | **y** | **z** |  |  |
| *Positive weight* | | | | | |
| **1** | 0 | 6 | 18 | 0.0350 | near left caudate |
| **2** | 0 | 9 | 15 | 0.0324 | near left caudate |
| **3** | 0 | 6 | 15 | 0.0301 | near left caudate |
| **4** | 0 | 3 | 18 | 0.0255 | near left caudate |
| **5** | 3 | -42 | 27 | 0.0196 | Right posterior cingulate gyrus |
| **6** | -3 | 9 | 6 | 0.0139 | near left caudate |
| **7** | -15 | -63 | 15 | 0.0132 | Left calcarine |
| **8** | 3 | -39 | 27 | 0.0129 | Right posterior cingulate gyrus |
| **9** | -3 | -84 | 45 | 0.0101 | near left cuneus |
| **10** | 0 | -27 | 30 | 0.00934 | near left median cingulate |
| *Negative weight* | | | | | |
| **1** | -42 | -75 | -21 | -0.0507 | Left cerebelum Crus1 |
| **2** | -18 | -15 | -18 | -0.0342 | Left hippocampus |
| **3** | 12 | -24 | -39 | -0.0323 | near right cerebelum 10 |
| **4** | 9 | -24 | -39 | -0.0284 | near right cerebelum 10 |
| **5** | 24 | 9 | -12 | -0.0256 | Right olfactory |
| **6** | 9 | -24 | -42 | -0.0206 | near right cerebelum 10 |
| **7** | -48 | -36 | -30 | -0.0186 | near left cerebelum Crus1 |
| **8** | 6 | -24 | -42 | -0.0168 | near right Cerebelum 9 |
| **9** | 27 | 9 | -12 | -0.0164 | Right olfactory |
| **10** | -3 | -6 | -12 | -0.0156 | near left hippocampus |

**STable 2c. Top 10 contributive ALFF regions and their discriminative weights on global signal not removed data**

| **Rank** | **MNI Coordinate** | | | **Weight** | **Region** |
| --- | --- | --- | --- | --- | --- |
|  | **x** | **y** | **z** |  |  |
| *Positive weight* | | | | | |
| **1** | -18 | -63 | 15 | 0.0241 | Left calcarine |
| **2** | 3 | -48 | 33 | 0.0219 | Right median cingulate |
| **3** | -12 | -45 | 33 | 0.0192 | Left median cingulate |
| **4** | -3 | 9 | 6 | 0.0182 | near left caudate |
| **5** | 0 | 6 | 18 | 0.0182 | near left caudate |
| **6** | -6 | 6 | 6 | 0.0180 | near left caudate |
| **7** | 0 | 12 | 12 | 0.0179 | near left caudate |
| **8** | 0 | 9 | 15 | 0.0172 | near left caudate |
| **9** | 0 | 3 | 18 | 0.0141 | near left caudate |
| **10** | 0 | 6 | 15 | 0.0126 | near left caudate |
| *Negative weight* | | | | | |
| **1** | -48 | -36 | -30 | -0.0407 | near left cerebelum Crus1 |
| **2** | 12 | -24 | -39 | -0.0326 | near right cerebelum 10 |
| **3** | -18 | -15 | -18 | -0.0284 | Left hippocampus |
| **4** | -51 | -39 | -30 | -0.0277 | Left cerebelum Crus1 |
| **5** | 24 | 9 | -12 | -0.0249 | Right olfactory |
| **6** | -3 | -21 | -42 | -0.0245 | near left Cerebelum 10 |
| **7** | -3 | -6 | -12 | -0.0233 | near left hippocampus |
| **8** | 27 | 9 | -12 | -0.0217 | Right olfactory |
| **9** | 6 | -24 | -42 | -0.0200 | near right Cerebelum 9 |
| **10** | 12 | -27 | -39 | -0.0195 | near right cerebelum 10 |

**STable 2d. Top 10 contributive ALFF regions and their discriminative weights on scrubbing data**

| **Rank** | **MNI Coordinate** | | | **Weight** | **Region** |
| --- | --- | --- | --- | --- | --- |
|  | **x** | **y** | **z** |  |  |
| *Positive weight* | | | | | |
| **1** | 3 | -36 | 48 | 0.0148 | Right median cingulate |
| **2** | -3 | -72 | 54 | 0.0144 | Left precuneus |
| **3** | 0 | -36 | 0 | 0.0133 | near vermis 3 |
| **4** | -3 | -81 | 45 | 0.0132 | Left precuneus |
| **5** | -3 | -81 | 48 | 0.0128 | Left precuneus |
| **6** | -15 | -63 | 15 | 0.0127 | Left calcarine |
| **7** | 0 | 3 | 60 | 0.0126 | Left supplementary motor area |
| **8** | 3 | -36 | 27 | 0.0125 | Right posterior cingulate gyrus |
| **9** | -60 | 6 | -3 | 0.0121 | Left superior temporal gyrus |
| **10** | 3 | -36 | 0 | 0.0148 | Right lingual gyrus |
| *Negative weight* | | | | | |
| **1** | 6 | -42 | 24 | -0.0170 | Right posterior cingulate gyrus |
| **2** | 27 | -3 | 0 | -0.0140 | Right putamen |
| **3** | 27 | -3 | 3 | -0.0108 | Right putamen |
| **4** | -24 | 0 | 0 | -0.0103 | Left Pallidum |
| **5** | -3 | -42 | 24 | -0.0100 | Left posterior cingulate gyrus |
| **6** | 27 | 0 | 3 | -0.00972 | Right putamen |
| **7** | -27 | -6 | 0 | -0.00921 | Left Pallidum |
| **8** | 3 | -42 | 24 | -0.00902 | Right posterior cingulate gyrus |
| **9** | 27 | 0 | 0 | -0.00887 | Right putamen |
| **10** | 0 | -33 | 30 | -0.00827 | Left posterior cingulate gyrus |

**STable 3a. Top 10 contributive GMV regions and their discriminative weights on 5-fold cross-validation**

| **Rank** | **MNI Coordinate** | | | **Weight** | **Region** |
| --- | --- | --- | --- | --- | --- |
|  | **x** | **y** | **z** |  |  |
| *Positive weight* | | | | | |
| **1** | 21 | 21 | 51 | 0.000763 | Right superior frontal gyrus |
| **2** | 39 | 12 | 45 | 0.000740 | Right middle frontal gyrus |
| **3** | -48 | -75 | -18 | 0.000736 | near left inferior occipital gyrus |
| **4** | -48 | -75 | -21 | 0.000707 | near left cerebelum Crus1 |
| **5** | 39 | 9 | 45 | 0.000699 | Right middle frontal gyrus |
| **6** | -51 | -75 | -18 | 0.000689 | near left inferior occipital gyrus |
| **7** | 15 | -66 | 51 | 0.000681 | Right superior parietal gyrus |
| **8** | -27 | -84 | 42 | 0.000679 | near left superior occipital gyrus |
| **9** | 42 | -72 | -48 | 0.000675 | Right cerebelum Crus2 |
| **10** | 45 | -72 | -48 | 0.000673 | Right cerebelum Crus2 |
| *Negative weight* | | | | | |
| **1** | -51 | 30 | 3 | -0.000620 | Left inferior frontal gyrus |
| **2** | -48 | 30 | 27 | -0.000619 | Left inferior frontal gyrus |
| **3** | -27 | 12 | -21 | -0.000597 | near left inferior frontal gyrus |
| **4** | -51 | 30 | 0 | -0.000577 | Left inferior frontal gyrus |
| **5** | -39 | -30 | 54 | -0.000576 | Left postcentral gyrus |
| **6** | 36 | 3 | 6 | -0.000576 | near right insula |
| **7** | -21 | 9 | -21 | -0.000569 | Left inferior frontal gyrus |
| **8** | -36 | -30 | 54 | -0.000569 | Left postcentral gyrus |
| **9** | -51 | 30 | 27 | -0.000566 | Left inferior frontal gyrus |
| **10** | -51 | 30 | 6 | -0.000565 | Left inferior frontal gyrus |

**STable 3b. Top 10 contributive GMV regions and their discriminative weights on 10-fold cross-validation**

| **Rank** | **MNI Coordinate** | | | **Weight** | **Region** |
| --- | --- | --- | --- | --- | --- |
|  | **x** | **y** | **z** |  |  |
| *Positive weight* | | | | | |
| **1** | 39 | 9 | 42 | 0.000766 | Right middle frontal gyrus |
| **2** | 39 | 12 | 45 | 0.000765 | Right middle frontal gyrus |
| **3** | 21 | 21 | 51 | 0.000759 | Right superior frontal gyrus |
| **4** | 39 | 9 | 45 | 0.000749 | Right middle frontal gyrus |
| **5** | 36 | 12 | 45 | 0.000727 | Right middle frontal gyrus |
| **6** | -48 | -75 | -18 | 0.000727 | near left inferior occipital gyrus |
| **7** | 21 | 21 | 48 | 0.000695 | Right superior frontal gyrus |
| **8** | 15 | -66 | 54 | 0.000684 | Right superior parietal gyrus |
| **9** | -15 | 45 | 45 | 0.000683 | Left superior frontal gyrus |
| **10** | 15 | -66 | 51 | 0.000682 | Right superior parietal gyrus |
| *Negative weight* | | | | | |
| **1** | -39 | -30 | 54 | -0.000688 | Left postcentral gyrus |
| **2** | -51 | 30 | 3 | -0.000687 | Left inferior frontal gyrus |
| **3** | -66 | -36 | 24 | -0.000661 | near left superior temporal gyrus |
| **4** | -51 | 30 | 27 | -0.000659 | Left inferior frontal gyrus |
| **5** | -36 | -30 | 54 | -0.000651 | Left postcentral gyrus |
| **6** | -12 | -18 | 3 | -0.000622 | Left thalamus |
| **7** | 36 | 0 | 9 | -0.000618 | near right insula |
| **8** | -51 | 30 | 0 | -0.000616 | Left inferior frontal gyrus |
| **9** | 36 | 3 | 6 | -0.000616 | near right insula |
| **10** | -36 | -30 | 57 | -0.000616 | Left postcentral gyrus |

**STable 4a. Top 10 contributive FA regions and their discriminative weights on 5-fold cross-validation**

| **Rank** | **MNI Coordinate** | | | **Weight** | **Region** |
| --- | --- | --- | --- | --- | --- |
|  | **x** | **y** | **z** |  |  |
| *Positive weight* | | | | | |
| **1** | 36 | 42 | -4 | 0.00128 | near right anterior corona radiata |
| **2** | -21 | 47 | 12 | 0.00122 | near left anterior corona radiata |
| **3** | 22 | 7 | 15 | 0.00117 | Right anterior limb of internal capsule |
| **4** | -21 | 46 | 12 | 0.00114 | near left anterior corona radiata |
| **5** | 15 | -7 | 54 | 0.00113 | near right superior corona radiata |
| **6** | 16 | -7 | 54 | 0.00113 | near right superior corona radiata |
| **7** | -34 | 23 | 19 | 0.00110 | near left anterior corona radiata |
| **8** | 16 | -8 | 54 | 0.00110 | near right superior corona radiata |
| **9** | 8 | -50 | -16 | 0.00109 | near right inferior cerebellar peduncle |
| *Negative weight* | | | | | |
| **10** | 3 | -19 | -6 | -0.00105 | near right cerebral peduncle |
| **1** | 3 | -18 | -5 | -0.000956 | near right cerebral peduncle |
| **2** | 3 | -19 | -5 | -0.000948 | near right cerebral peduncle |
| **3** | 3 | -18 | -6 | -0.000942 | near right cerebral peduncle |
| **4** | 3 | -20 | -6 | -0.000888 | near right cerebral peduncle |
| **5** | 7 | 25 | -5 | -0.000877 | near genu of corpus callosum |
| **6** | 35 | -20 | -2 | -0.000875 | Right external capsule |
| **7** | 3 | -20 | -5 | -0.000831 | near right cerebral peduncle |
| **8** | -12 | 29 | -5 | -0.000820 | Genu of corpus callosum |
| **9** | 3 | -17 | -5 | -0.000811 | near right cerebral peduncle |
| **10** | 3 | -19 | -6 | -0.000956 | near right cerebral peduncle |

**STable 4b. Top 10 contributive FA regions and their discriminative weights on 10-fold cross-validation**

| **Rank** | **MNI Coordinate** | | | **Weight** | **Region** |
| --- | --- | --- | --- | --- | --- |
|  | **x** | **y** | **z** |  |  |
| *Positive weight* | | | | | |
| **1** | 36 | 42 | -4 | 0.00150 | near right anterior corona radiata |
| **2** | 35 | 42 | -4 | 0.00141 | near right anterior corona radiata |
| **3** | 35 | 42 | -3 | 0.00135 | near right anterior corona radiata |
| **4** | 36 | 42 | -5 | 0.00125 | near right anterior corona radiata |
| **5** | 21 | 7 | 15 | 0.00123 | Right anterior limb of internal capsule |
| **6** | 15 | -8 | 54 | 0.00122 | near right superior corona radiata |
| **7** | 22 | 7 | 15 | 0.00118 | Right anterior limb of internal capsule |
| **8** | 15 | -7 | 54 | 0.00118 | near right superior corona radiata |
| **9** | 36 | 43 | -4 | 0.00118 | near right anterior corona radiata |
| **10** | 23 | 10 | 16 | 0.00117 | Right anterior limb of internal capsule |
| *Negative weight* | | | | | |
| **1** | 3 | -19 | -6 | -0.00107 | near right cerebral peduncle |
| **2** | 3 | -18 | -6 | -0.00100 | near right cerebral peduncle |
| **3** | 35 | -21 | -2 | -0.000974 | Right retrolenticular part of internal capsule |
| **4** | 3 | -18 | -5 | -0.000965 | near right cerebral peduncle |
| **5** | 3 | -19 | -5 | -0.000964 | near right cerebral peduncle |
| **6** | 7 | 24 | -5 | -0.000940 | near genu of corpus callosum |
| **7** | 9 | 26 | -5 | -0.000896 | Genu of corpus callosum |
| **8** | 3 | -20 | -6 | -0.000893 | near right cerebral peduncle |
| **9** | 36 | -22 | -2 | -0.000888 | near right retrolenticular part of internal capsule |
| **10** | 3 | -17 | -5 | -0.000887 | near right cerebral peduncle |

**STable 4c. Top 10 contributive FA regions and their discriminative weights on TBSS data**

| **Rank** | **MNI Coordinate** | | | **Weight** | **Region** |
| --- | --- | --- | --- | --- | --- |
|  | **x** | **y** | **z** |  |  |
| *Positive weight* | | | | | |
| **1** | -44 | 28 | 9 | 0.00176 | near left anterior corona radiata |
| **2** | -43 | 29 | 9 | 0.00176 | near left anterior corona radiata |
| **3** | -43 | 28 | 8 | 0.00167 | near left anterior corona radiata |
| **4** | 11 | -19 | -28 | 0.00165 | Middle cerebellar peduncle |
| **5** | -32 | 3 | -32 | 0.00159 | near left uncinate fasciculus |
| **6** | 8 | -19 | -28 | 0.00156 | Right corticospinal tract |
| **7** | -43 | 28 | 9 | 0.00154 | near left anterior corona radiata |
| **8** | -43 | 29 | 8 | 0.00152 | near left anterior corona radiata |
| **9** | -16 | 11 | 56 | 0.00144 | near left superior corona radiata |
| **10** | 21 | -53 | -20 | 0.00143 | near middle cerebellar peduncle |
| *Negative weight* | | | | | |
| **1** | -11 | 15 | -9 | -0.00177 | near genu of corpus callosum |
| **2** | 16 | 9 | -8 | -0.00127 | near right anterior limb of internal capsule |
| **3** | 27 | 39 | -2 | -0.00126 | near right anterior corona radiata |
| **4** | 26 | 39 | -2 | -0.00121 | near right anterior corona radiata |
| **5** | -34 | -57 | 18 | -0.00121 | near left posterior thalamic radiation |
| **6** | -18 | 7 | -6 | -0.00119 | near left anterior limb of internal capsule |
| **7** | 16 | 15 | -9 | -0.00117 | near right external capsule |
| **8** | -24 | 4 | -12 | -0.00117 | near left external capsule |
| **9** | 26 | 39 | -3 | -0.00114 | near right anterior corona radiata |
| **10** | 15 | 11 | -12 | -0.00113 | near right external capsule |

**STable 5. Results of tri-modality prediction model on visual working memory, fluid intelligence and emotional regulation**

| **Cognitive function** | **r value** | **Number of selected features** | | |
| --- | --- | --- | --- | --- |
|  |  | **ALFF** | **GMV** | **FA** |
| **Visual working memory** | 0.401 | 118 | 39366 | 19918 |
| **Fluid intelligence** | 0.656 | 1243 | 52948 | 44648 |
| **Emotional regulation^*^** | 0.085 | 78 | 463 | 119 |

r value: Pearson correlation between the predicted and the observed visual working memory scores; number of selected features: the average number of selected features across all the 547 folds in the cross-validation procedure.

**^*^** Pearson correlation feature selection p value is set to 0.001, because original p value used in main text is too stringent.

**STable 6a. Top 10 contributive ALFF regions and their discriminative weights of emotional regulation**

| **Rank** | **MNI Coordinate** | | | **Weight** | **Region** | | |
| --- | --- | --- | --- | --- | --- | --- | --- |
|  | **x** | **y** | **z** |  |  |  |  |
| *Positive weight* | | | | | | |  |
| **1** | 39 | 57 | 18 | 0.108 | Right middle frontal gyrus | | |
| **2** | -66 | -27 | 33 | 0.0928 | Left supramarginal gyrus | | |
| **3** | 24 | -78 | -3 | 0.0925 | Right fusiform | | |
| **4** | -66 | -30 | 33 | 0.0819 | near left supramarginal gyrus | | |
| **5** | 15 | 63 | -18 | 0.0585 | near right superior frontal medial gyrus | | |
| **6** | -3 | -96 | 24 | 0.0575 | Left cuneus | | |
| **7** | 66 | -21 | 33 | 0.0460 | Right supramarginal gyrus | | |
| **8** | 66 | -21 | 36 | 0.0446 | Right supramarginal gyrus | | |
| **9** | -33 | -93 | 9 | 0.0424 | Left middle occipital gyrus | | |
| **10** | 69 | -21 | 33 | 0.0316 | near right supramarginal gyrus | | |
| *Negative weight* | | | | | |  |  |
| **1** | -42 | -21 | -6 | -0.0608 | near left superior temporal gyrus | | |
| **2** | -33 | -42 | -12 | -0.0606 | Left fusiform | | |
| **3** | -12 | -3 | 48 | -0.0562 | Left supplementary motor area | | |
| **4** | 27 | -42 | 63 | -0.0559 | Right postcentral gyrus | | |
| **5** | 9 | -63 | 54 | -0.0553 | Right precuneus | | |
| **6** | -15 | 54 | 27 | -0.0540 | Left superior frontal gyrus | | |
| **7** | -57 | 3 | -24 | -0.0529 | Left middle temporal gyrus | | |
| **8** | -36 | -15 | -9 | -0.0514 | near left hippocampus | | |
| **9** | -48 | -51 | -18 | -0.0480 | Left inferior temporal gyrus | | |
| **10** | -57 | 6 | -24 | -0.0471 | Left middle temporal gyrus | | |

**STable 6b. Top 10 contributive ALFF regions and their discriminative weights of fluid intelligence**

| **Rank** | **MNI Coordinate** | | | **Weight** | **Region** | | |
| --- | --- | --- | --- | --- | --- | --- | --- |
|  | **x** | **y** | **z** |  |  |  |  |
| *Positive weight* | | | | | | |  |
| **1** | -12 | -27 | -27 | 0.181 | near left Cerebelum 4 5 | | |
| **2** | -12 | -63 | 18 | 0.110 | Left calcarine | | |
| **3** | 45 | -36 | 60 | 0.110 | Right postcentral gyrus | | |
| **4** | -30 | -33 | -39 | 0.106 | Left cerebelum 6 | | |
| **5** | 9 | -75 | 54 | 0.105 | Right precuneus | | |
| **6** | 0 | 48 | -18 | 0.104 | Left rectus | | |
| **7** | -3 | 9 | 6 | 0.103 | near left caudate | | |
| **8** | 0 | 30 | 45 | 0.0975 | Left superior frontal medial gyrus | | |
| **9** | -24 | 12 | -21 | 0.0960 | Left inferior frontal gyrus | | |
| **10** | 0 | 33 | 42 | 0.0949 | Left superior frontal medial gyrus | | |
| *Negative weight* | | | | | |  |  |
| **1** | -33 | 45 | -6 | -0.194 | Left medial orbital frontal gyrus | | |
| **2** | 33 | -81 | -15 | -0.138 | Right inferior occipital gyrus | | |
| **3** | 30 | 54 | -12 | -0.128 | Right superior frontal medial gyrus | | |
| **4** | 36 | -66 | -57 | -0.127 | Right cerebelum 8 | | |
| **5** | 21 | 3 | -9 | -0.127 | near right amygdala | | |
| **6** | 27 | 18 | -21 | -0.123 | Right inferior frontal gyrus | | |
| **7** | 30 | 21 | -21 | -0.118 | Right inferior frontal gyrus | | |
| **8** | 42 | -6 | 0 | -0.113 | Right insula | | |
| **9** | 21 | 0 | -6 | -0.112 | near right pallidum | | |
| **10** | 30 | -54 | 0 | -0.112 | near right fusiform | | |

**STable 7a. Top 10 contributive GMV regions and their discriminative weights of emotional regulation**

| **Rank** | **MNI Coordinate** | | | **Weight** | **Region** |
| --- | --- | --- | --- | --- | --- |
|  | **x** | **y** | **z** |  |  |
| *Positive weight* | | | | | |
| **1** | 36 | -78 | 3 | 0.0102 | Right middle occipital gyrus |
| **2** | 24 | -3 | -51 | 0.00908 | near right fusiform |
| **3** | 54 | -18 | 24 | 0.00871 | Right rolandic operculum |
| **4** | 36 | -81 | 3 | 0.00780 | Right middle occipital gyrus |
| **5** | 54 | -15 | 24 | 0.00779 | Right rolandic operculum |
| **6** | 21 | -6 | 69 | 0.00762 | Right superior frontal gyrus |
| **7** | 3 | -21 | -30 | 0.00761 | near right Cerebelum 3 |
| **8** | 21 | -6 | 72 | 0.00744 | Right superior frontal gyrus |
| **9** | 6 | -21 | -27 | 0.00741 | near right Cerebelum 3 |
| **10** | 27 | -3 | -51 | 0.00714 | near right fusiform |
| *Negative weights* | | | | | |
| **1** | -6 | 9 | 48 | -0.00663 | Left supplementary motor area |
| **2** | -6 | 9 | 51 | -0.00625 | Left supplementary motor area |
| **3** | -3 | 9 | 51 | -0.00619 | Left supplementary motor area |
| **4** | -30 | -75 | 36 | -0.00570 | Left middle occipital gyrus |
| **5** | -27 | -72 | 39 | -0.00562 | Left superior occipital gyrus |
| **6** | -3 | 9 | 48 | -0.00533 | Left supplementary motor area |
| **7** | -6 | 6 | 48 | -0.00505 | Left supplementary motor area |
| **8** | -6 | 6 | 51 | -0.00497 | Left supplementary motor area |
| **9** | -27 | -72 | 36 | -0.00483 | Left middle occipital gyrus |
| **10** | -27 | -75 | 36 | -0.00432 | Left middle occipital gyrus |

**STable 7b. Top 10 contributive GMV regions and their discriminative weights of fluid intelligence**

| **Rank** | **MNI Coordinate** | | | **Weight** | **Region** |
| --- | --- | --- | --- | --- | --- |
|  | **x** | **y** | **z** |  |  |
| *Positive weight* | | | | | |
| **1** | 30 | -72 | 27 | 0.00619 | Right middle occipital gyrus |
| **2** | 30 | -72 | 24 | 0.00616 | Right middle occipital gyrus |
| **3** | 42 | -63 | 3 | 0.00596 | Right middle temporal gyrus |
| **4** | 27 | -72 | 27 | 0.00562 | Right middle occipital gyrus |
| **5** | 42 | -60 | 3 | 0.00560 | Right middle temporal gyrus |
| **6** | 30 | -69 | 27 | 0.00556 | Right middle occipital gyrus |
| **7** | 27 | -69 | 27 | 0.00552 | Right superior occipital gyrus |
| **8** | 42 | -60 | 0 | 0.00552 | Right middle temporal gyrus |
| **9** | -51 | -27 | -9 | 0.00533 | Left middle temporal gyrus |
| **10** | 42 | -63 | 0 | 0.00533 | Right middle temporal gyrus |
| *Negative weights* | | | | | |
| **1** | -18 | -27 | 27 | -0.00730 | near left caudate |
| **2** | -18 | -24 | 27 | -0.00678 | near left caudate |
| **3** | -18 | -21 | 27 | -0.00542 | near left caudate |
| **4** | -30 | -45 | 66 | -0.00533 | Left superior parietal gyrus |
| **5** | -30 | -45 | 69 | -0.00515 | Left superior parietal gyrus |
| **6** | -54 | -24 | 27 | -0.00500 | Left supramarginal gyrus |
| **7** | -27 | -42 | 69 | -0.00484 | Left postcentral gyrus |
| **8** | -15 | -24 | 27 | -0.00480 | near left caudate |
| **9** | -27 | -45 | 69 | -0.00478 | Left superior parietal gyrus |
| **10** | -54 | -27 | 27 | -0.00476 | Left supramarginal gyrus |

**STable 8a. Top 10 contributive FA regions and their discriminative weights of emotional regulation**

| **Rank** | **MNI Coordinate** | | | **Weight** | **Region** |
| --- | --- | --- | --- | --- | --- |
|  | **x** | **y** | **z** |  |  |
| *Positive weight* | | | | | |
| **1** | 31 | 18 | 51 | 0.0634 | near right superior corona radiata |
| **2** | 34 | 37 | 25 | 0.0424 | near right anterior corona radiata |
| **3** | 34 | 36 | 25 | 0.0423 | near right anterior corona radiata |
| **4** | 49 | -23 | -22 | 0.0409 | near right near sagittal stratum |
| **5** | 35 | 37 | 26 | 0.0393 | near right anterior corona radiata |
| **6** | -7 | -44 | 65 | 0.0313 | near left posterior corona radiata |
| **7** | -29 | -6 | -43 | 0.0312 | near left cingulum(hippocampus) |
| **8** | -30 | -6 | -43 | 0.0294 | near left cingulum(hippocampus) |
| **9** | -15 | -44 | -19 | 0.0280 | near left inferior cerebellar peduncle |
| **10** | 35 | 4 | 2 | 0.0267 | near right external capsule |
| *Negative weight* | | | | | |
| **1** | 30 | -5 | 17 | -0.0193 | Right external capsule |
| **2** | -28 | -10 | 18 | -0.0165 | Left external capsule |
| **3** | 29 | -6 | 18 | -0.0156 | Right external capsule |
| **4** | 30 | -5 | 18 | -0.0145 | Right external capsule |
| **5** | 30 | -6 | 17 | -0.0140 | Right external capsule |
| **6** | 45 | -45 | 4 | -0.00995 | near right superior longitudinal fasciculus |
| **7** | 45 | -44 | 3 | -0.00827 | near right superior longitudinal fasciculus |
| **8** | -28 | -11 | 19 | -0.00810 | Left superior corona radiata |
| **9** | -28 | -11 | 18 | -0.00792 | Left external capsule |
| **10** | 19 | -1 | 43 | -0.00714 | near right superior corona radiata |

**STable 8b. Top 10 contributive FA regions and their discriminative weights of fluid intelligence**

| **Rank** | **MNI Coordinate** | | | **Weight** | **Region** |
| --- | --- | --- | --- | --- | --- |
|  | **x** | **y** | **z** |  |  |
| *Positive weight* | | | | | |
| **1** | -30 | 43 | 13 | 0.0108 | near left anterior corona radiata |
| **2** | -30 | 44 | 13 | 0.0101 | near left anterior corona radiata |
| **3** | -30 | 43 | 12 | 0.0100 | near left anterior corona radiata |
| **4** | -30 | 42 | 13 | 0.00972 | near left anterior corona radiata |
| **5** | -8 | 20 | -11 | 0.00956 | near genu of corpus callosum |
| **6** | -30 | 43 | 14 | 0.00925 | near left anterior corona radiata |
| **7** | -8 | 21 | -11 | 0.00922 | near genu of corpus callosum |
| **8** | -30 | 42 | 14 | 0.00909 | near left anterior corona radiata |
| **9** | -31 | 44 | 13 | 0.00889 | near left anterior corona radiata |
| **10** | -7 | 20 | -11 | 0.00873 | near genu of corpus callosum |
| *Negative weight* | | | | | |
| **1** | -29 | -23 | -9 | -0.00696 | Left Fornix (cres) / Stria terminalis |
| **2** | -5 | 25 | 0 | -0.00691 | Genu of corpus callosum |
| **3** | -10 | 19 | -6 | -0.00647 | near genu of corpus callosum |
| **4** | -4 | 25 | 0 | -0.00646 | Genu of corpus callosum |
| **5** | 43 | -25 | 4 | -0.00636 | near right retrolenticular part of internal capsule |
| **6** | 14 | -24 | -1 | -0.00630 | near right cerebral peduncle |
| **7** | -4 | 24 | 0 | -0.00623 | Genu of corpus callosum |
| **8** | -40 | 12 | 16 | -0.00614 | near left superior longitudinal fasciculus |
| **9** | 3 | 24 | -1 | -0.00612 | Genu of corpus callosum |
| **10** | -29 | -22 | -9 | -0.00612 | Left fornix (cres) / Stria terminalis |

**Figures**


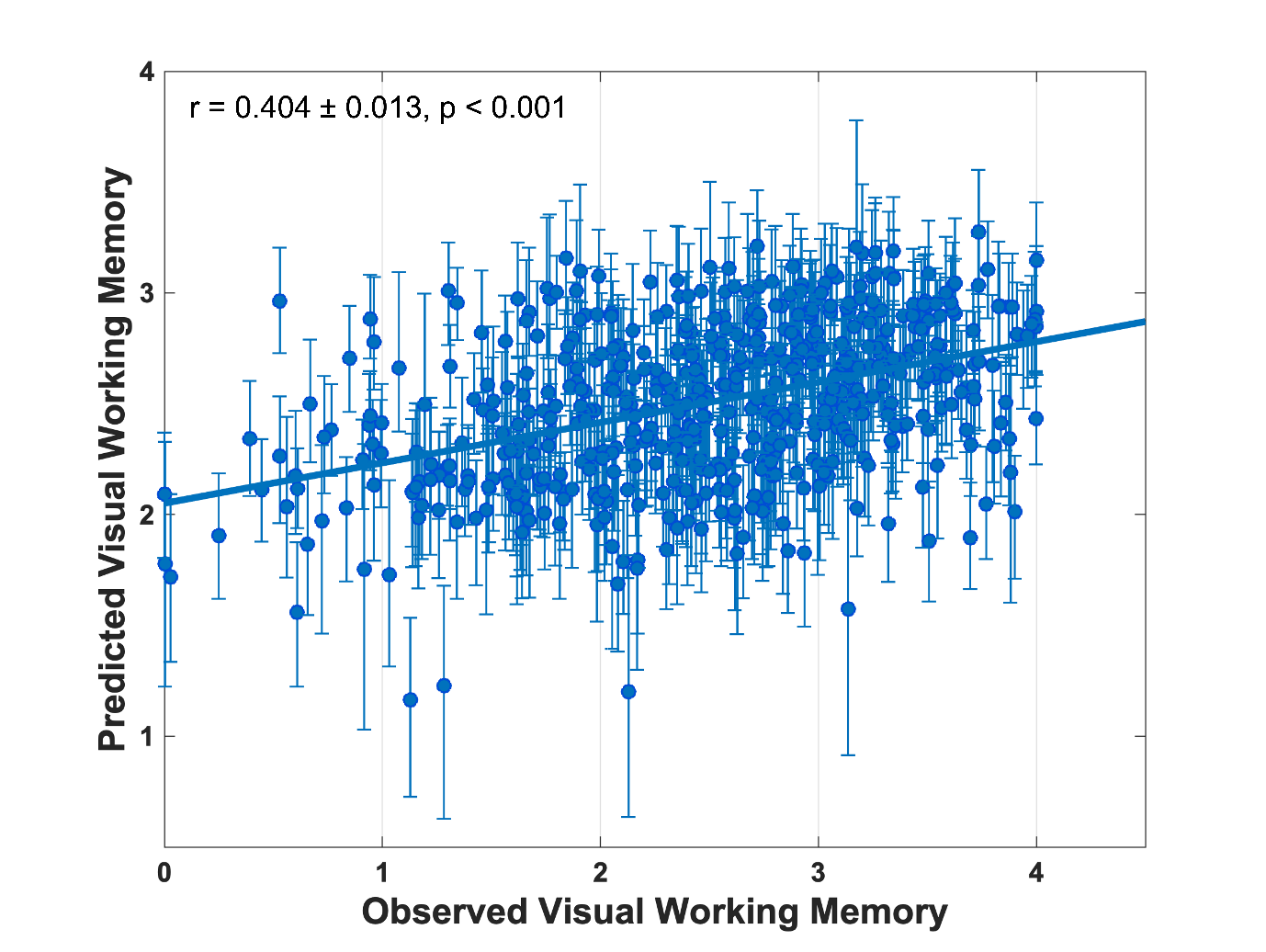


**SFigure 1a.** Validation results: correlation between the observed VWM score and the VWM score predicted by the bagging model of three modalities in the case of 5-fold cross-validation.


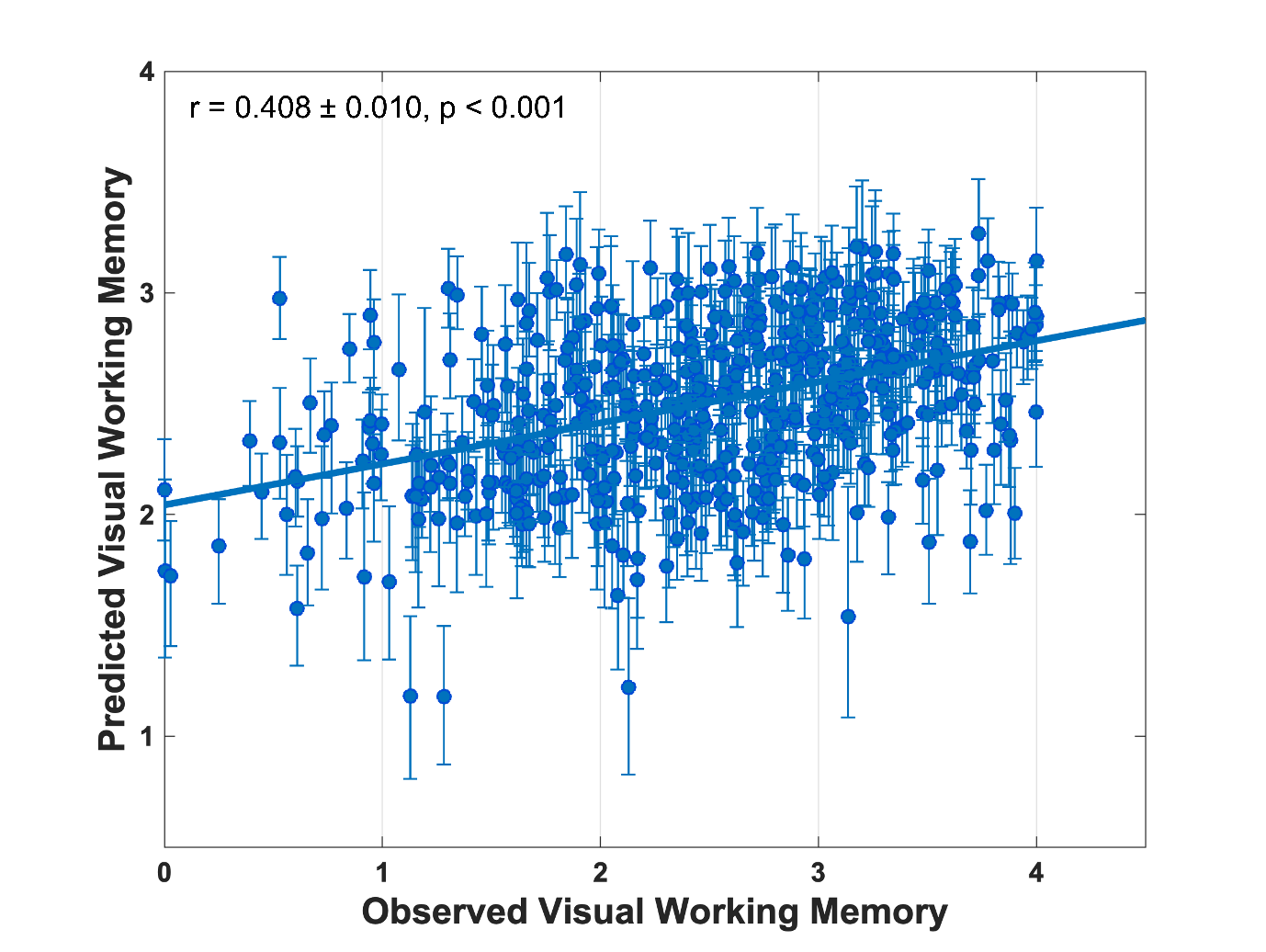


**SFigure 1b.** Validation results: correlation between the observed VWM score and the VWM score predicted by the bagging model of three modalities in the case of 10-fold cross-validation.


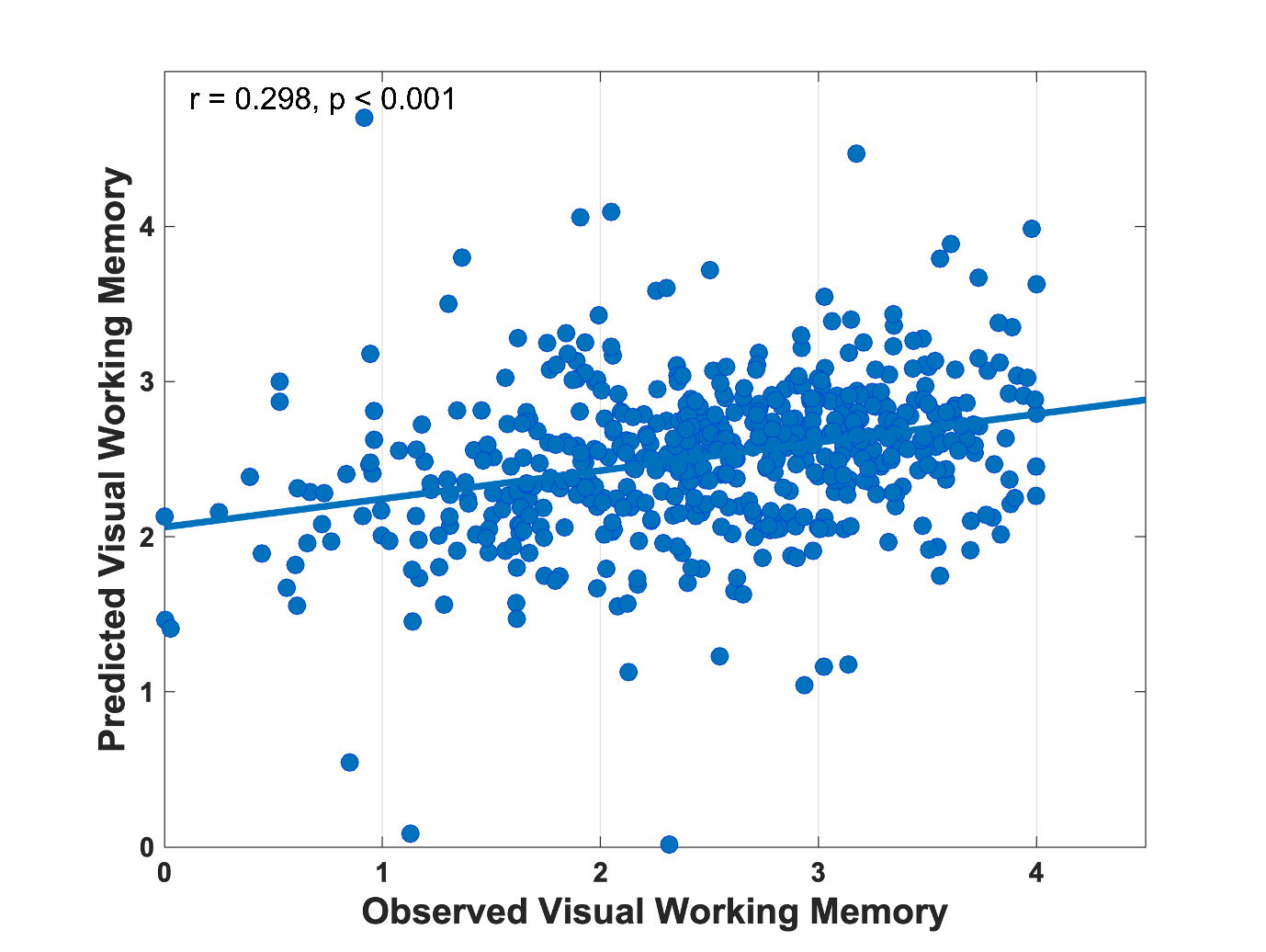


**SFigure 1c.** Validation results: correlation between the observed VWM score and the VWM score predicted by the bagging model of three modalities in the case that the prediction model was built from linear regression.


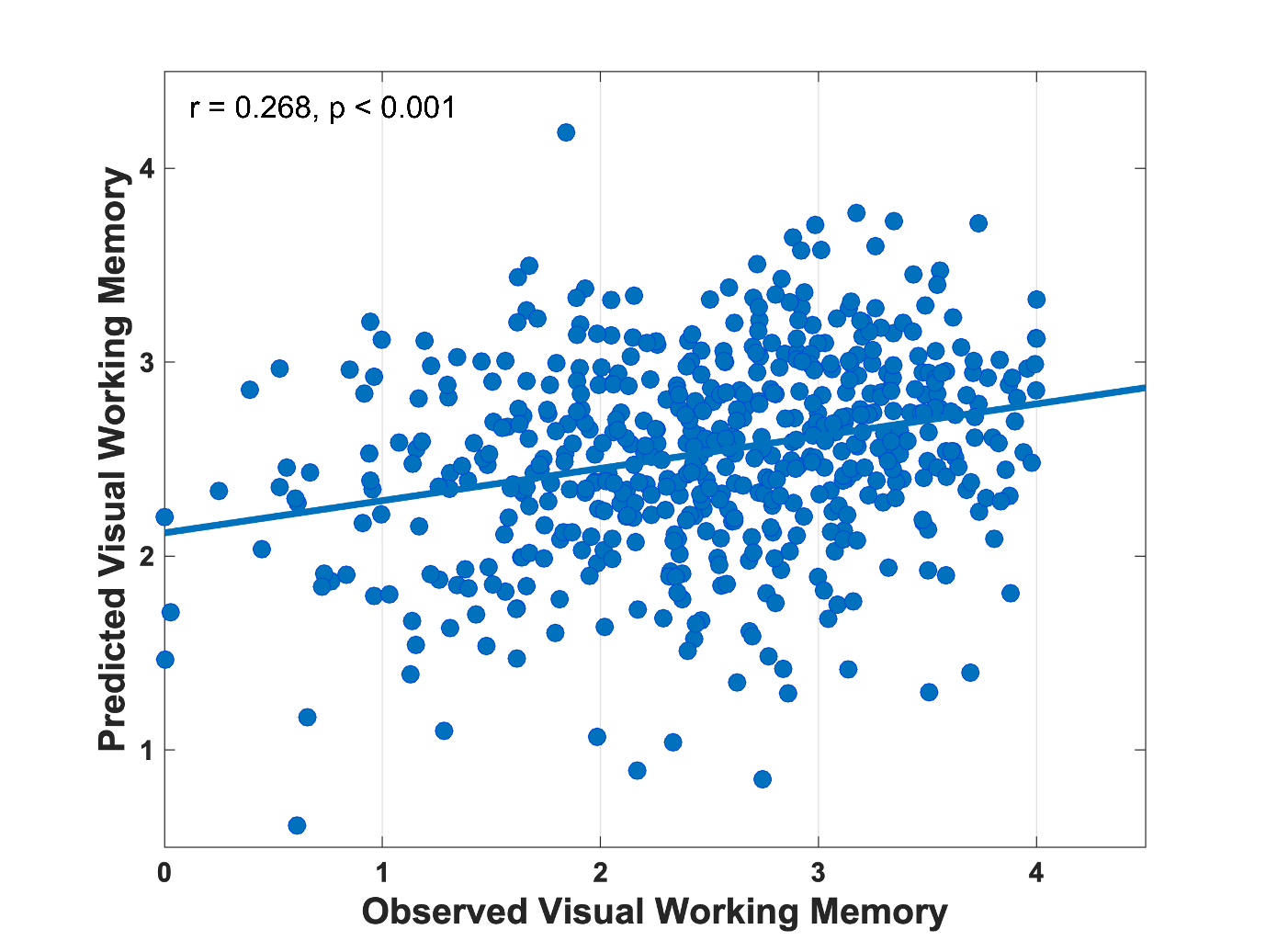


**SFigure 1d.** Validation results: correlation between the observed VWM score and the VWM score predicted by the bagging model of three modalities in the case that the prediction model was built from support vector regression.


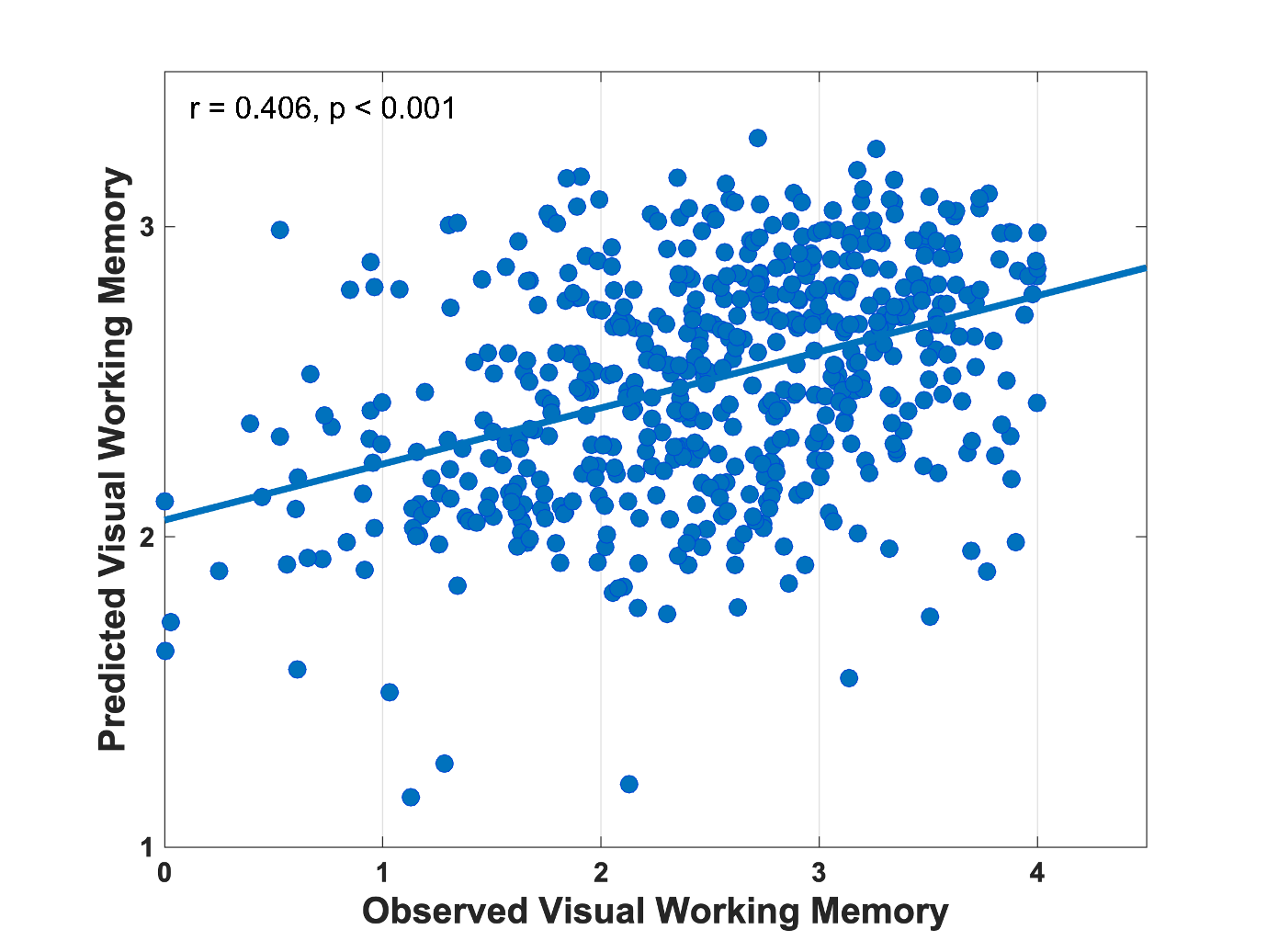


**SFigure 1e.** Validation results: correlation between the observed VWM score and the VWM score predicted by the bagging model of the three modalities in the case that the data was preprocessed without global signal removal.


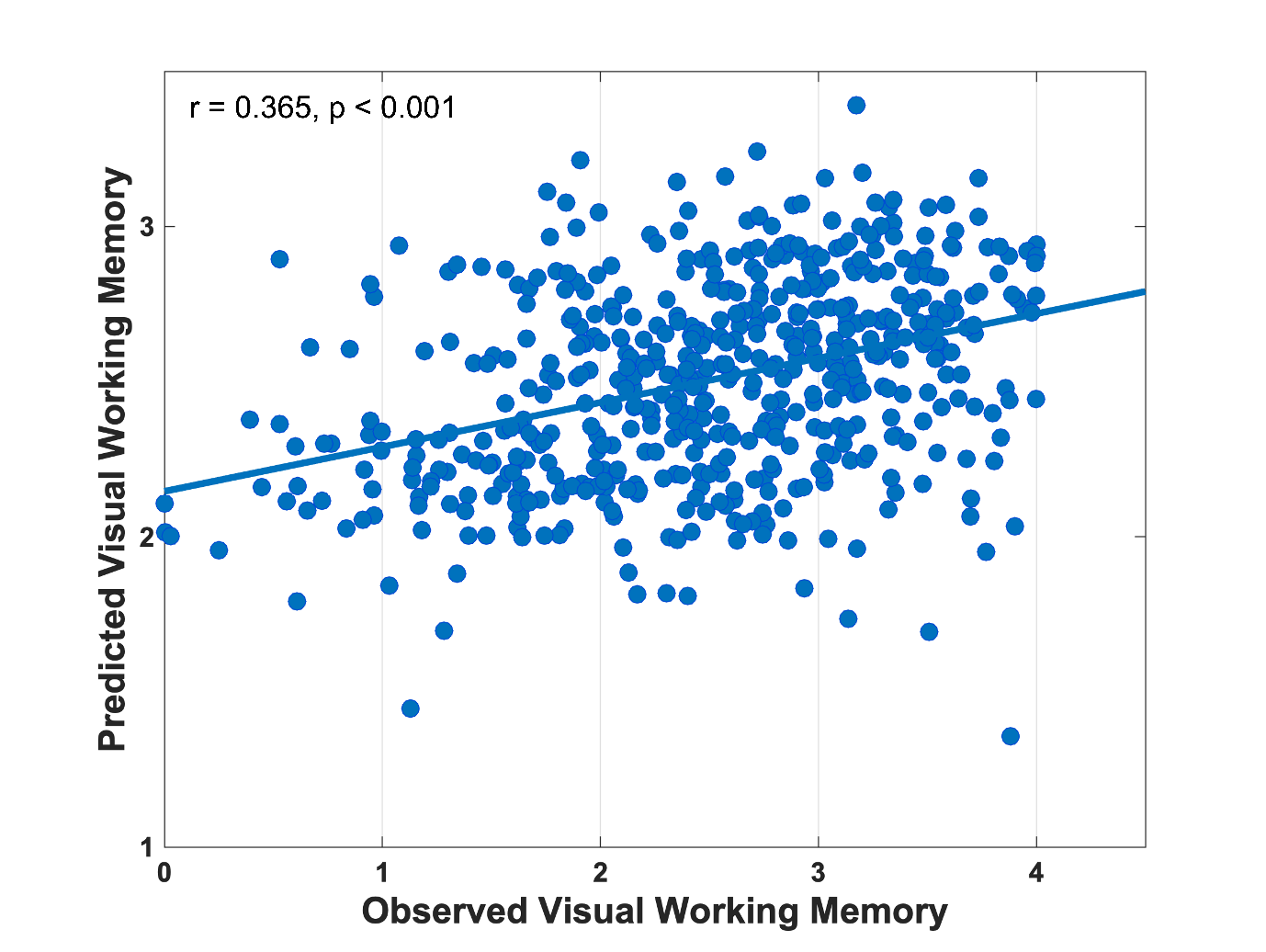


**SFigure 1f.** Validation results: correlation between the observed VWM score and the VWM score predicted by the bagging model of three modalities in the case that the data was preprocessed with motion scrubbing.


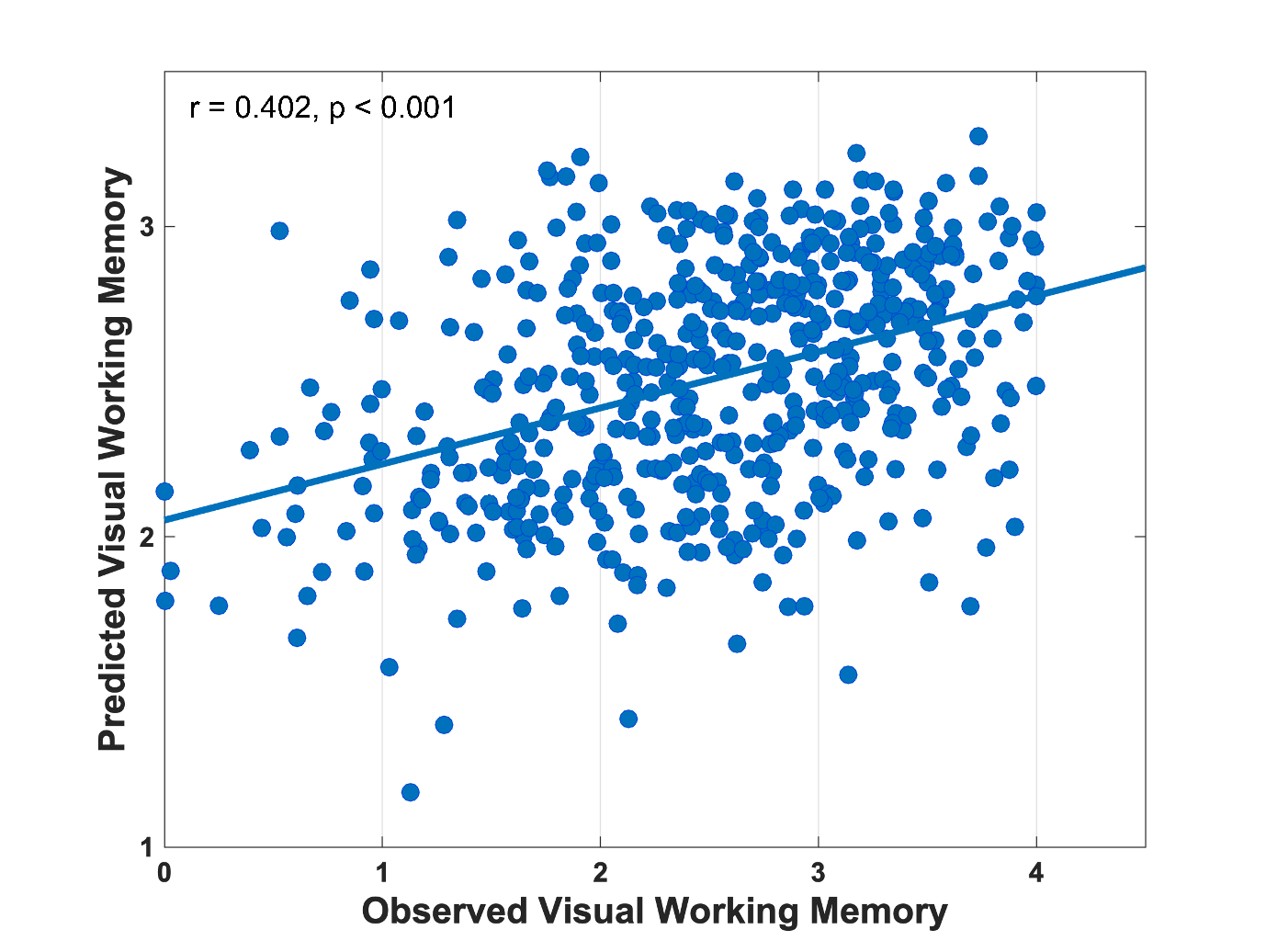


**SFigure 1g.** Validation results: correlation between the observed VWM score and the VWM score predicted by the bagging model of three modalities in the case that the data was preprocessed with TBSS.


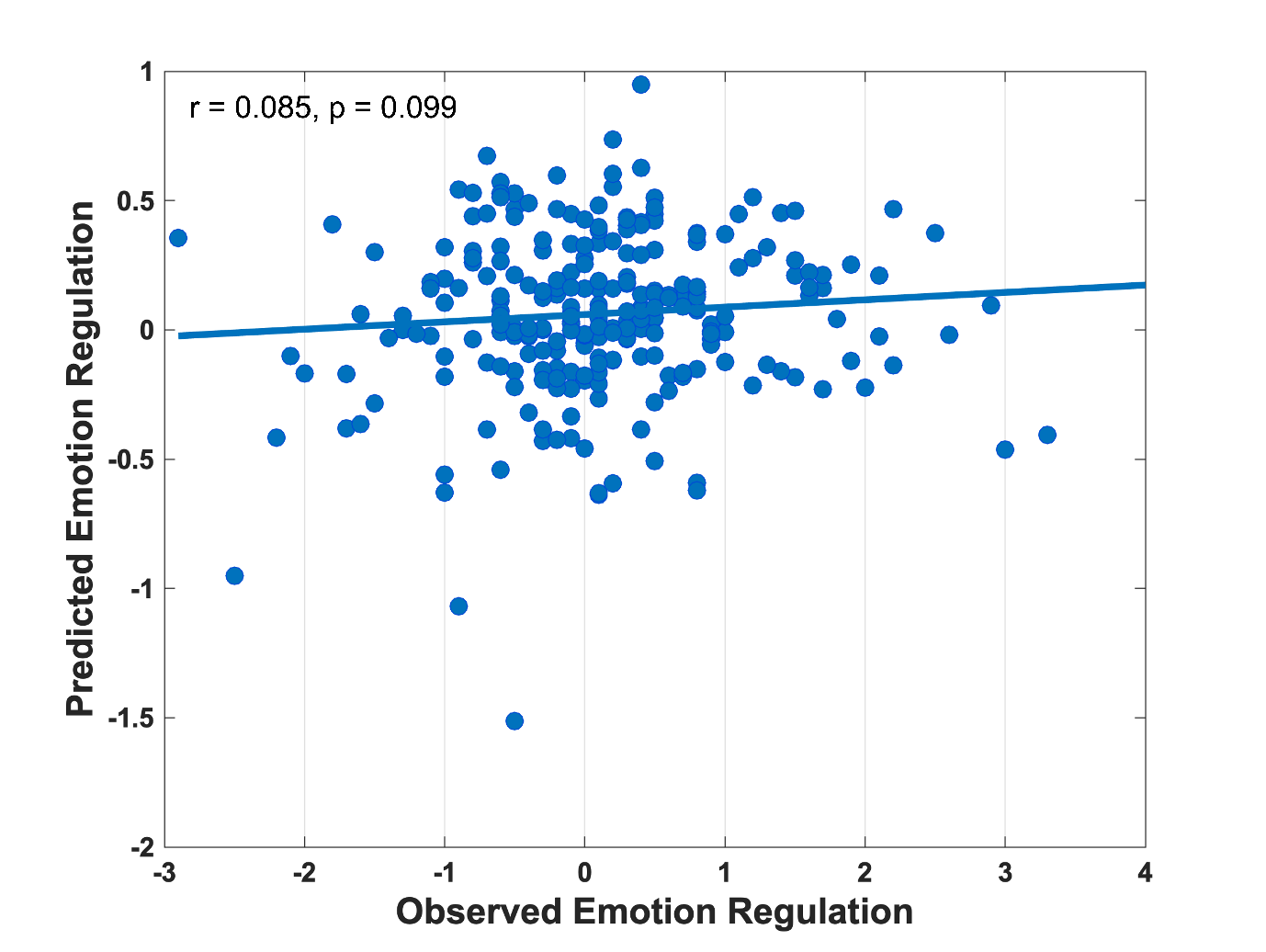


**SFigure 1h.** Validation results: correlation between the observed VWM score and the VWM score predicted by the bagging model of three modalities in the case of emotional regulation prediction.


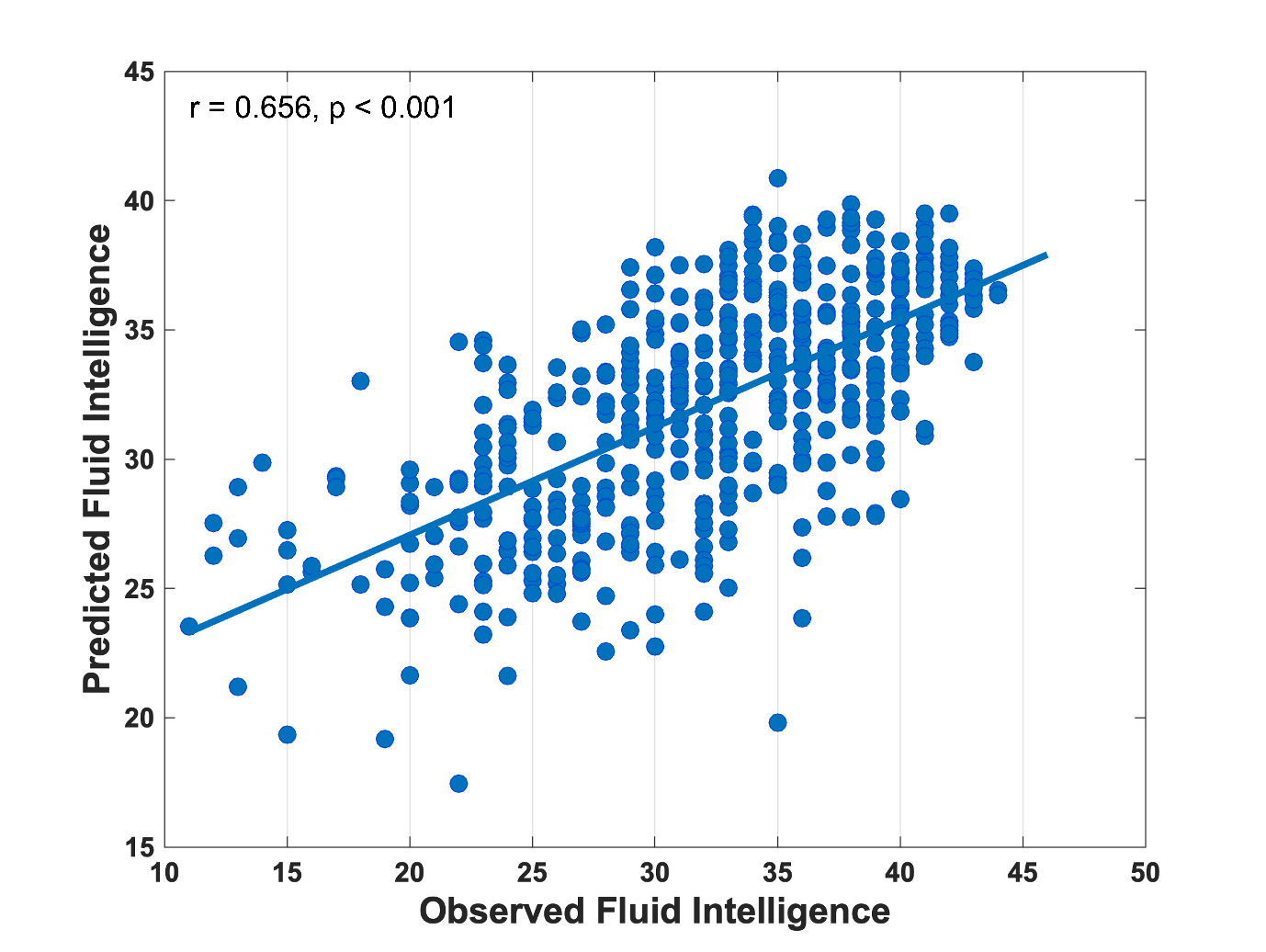


**SFigure 1i.** Validation results: correlation between the observed VWM score and the VWM score predicted by the bagging model of three modalities in the case of fluid intelligence prediction.


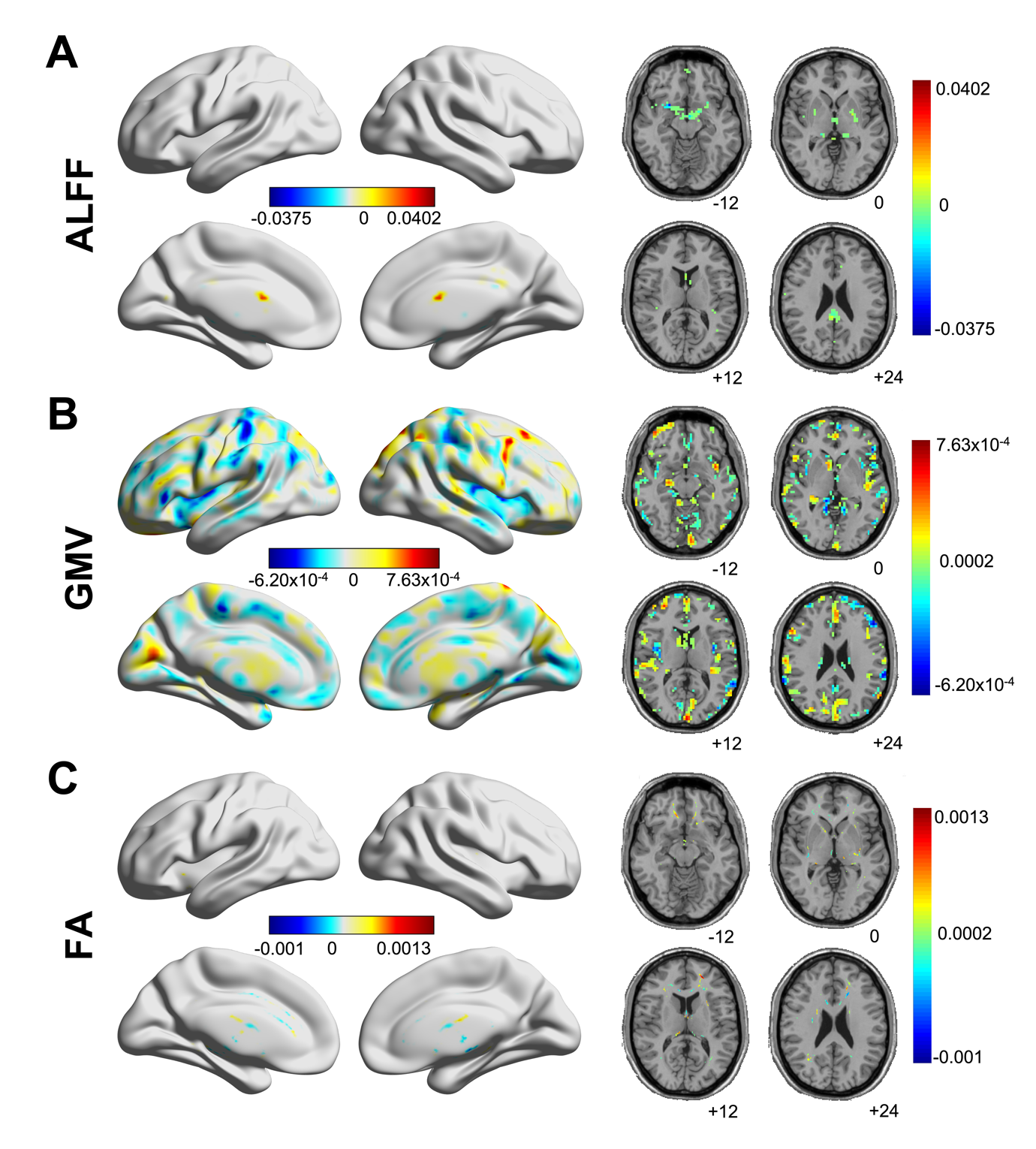


**SFigure 2a.** Validation results: discriminative voxel-wise weight of (A) ALFF, (B) GMV, and (C) FA in the case that the prediction model was built from 5-fold cross-validation. In all the three maps, warm color indicates positive weights, whilst cold color indicates negative weights. Darker color indicates larger absolute values of weights.


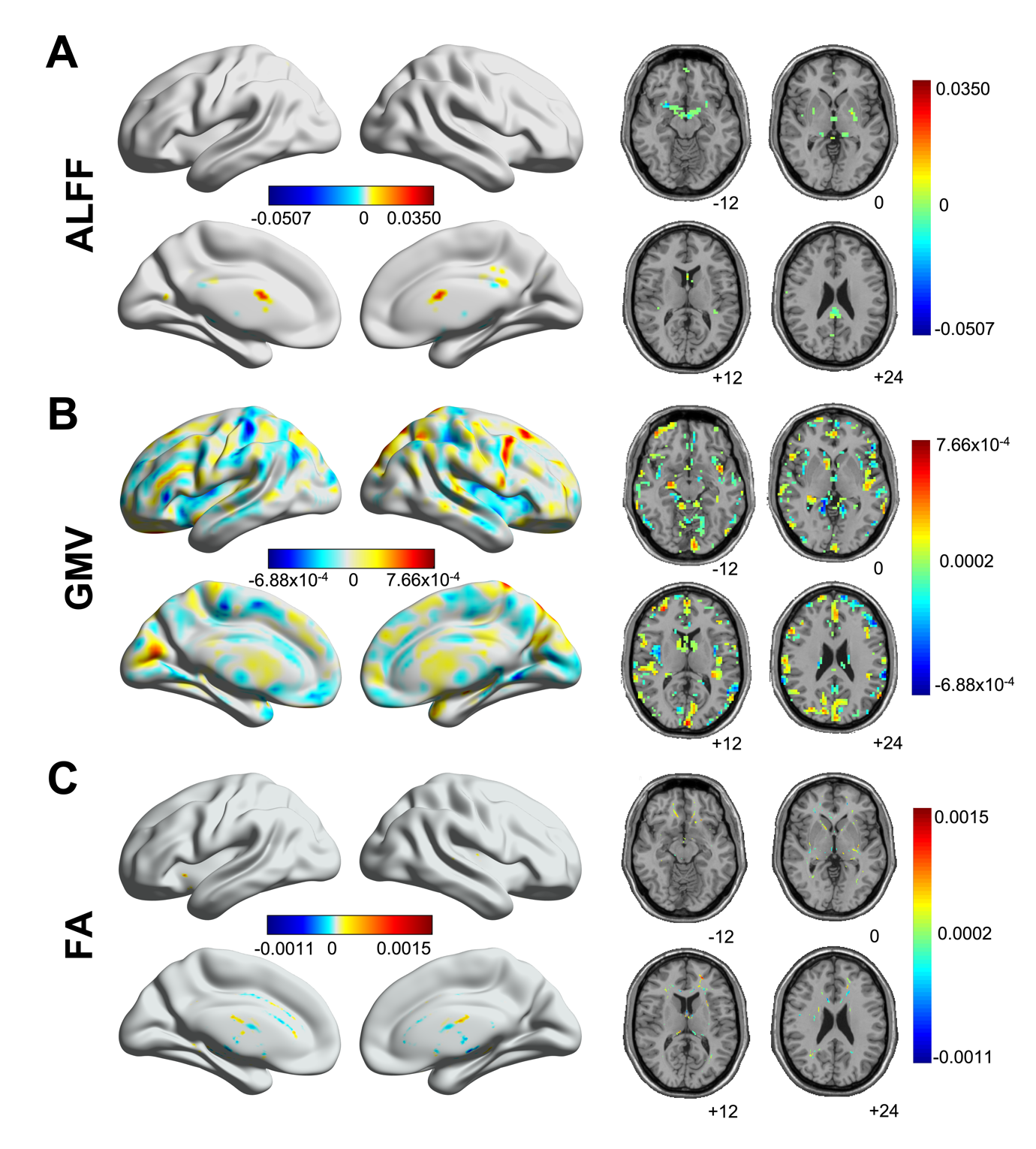


**SFigure 2b.** Validation results: discriminative voxel-wise weight of (A) ALFF, (B) GMV, and (C) FA in the case that the prediction model was built from 10-fold cross-validation. In all the three maps, warm color indicates positive weights, whilst cold color indicates negative weights. Darker color indicates larger absolute values of weights.


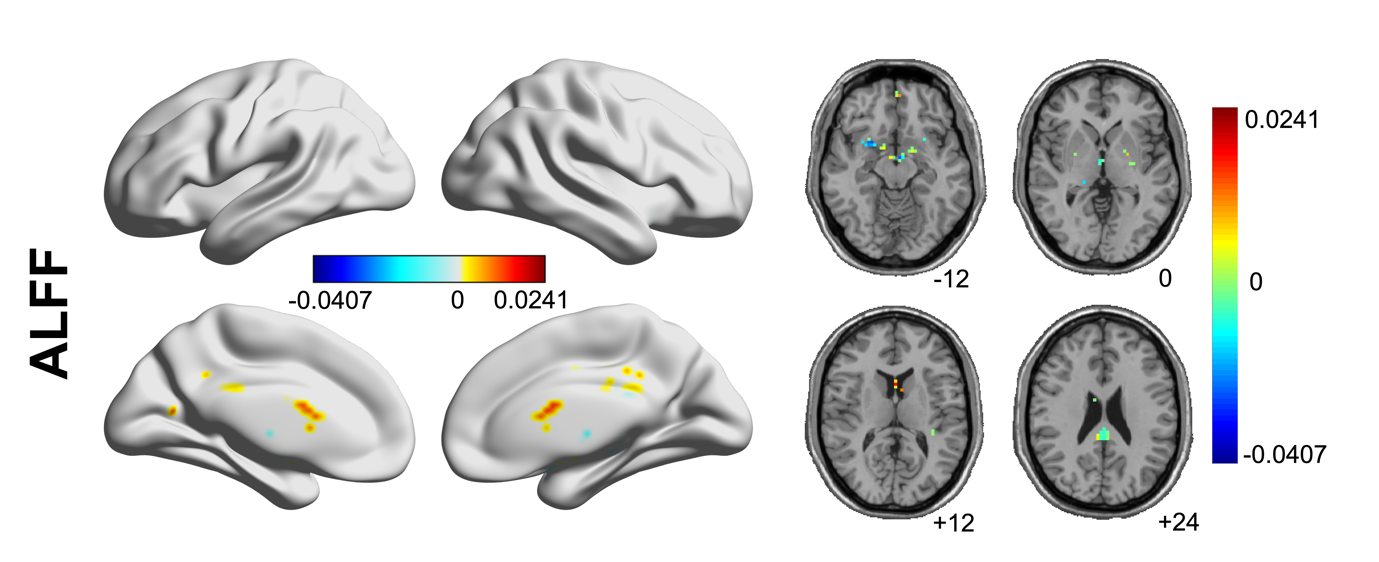


**SFigure 2c.** Validation results: discriminative voxel-wise weight of ALFF in the case that the data was preprocessed without global signal removal. The maps of GMV and FA were not shown as their extraction would not be influenced in this case. Warm color indicates positive weights, whilst cold color indicates negative weights. Darker color indicates larger absolute values of weights.


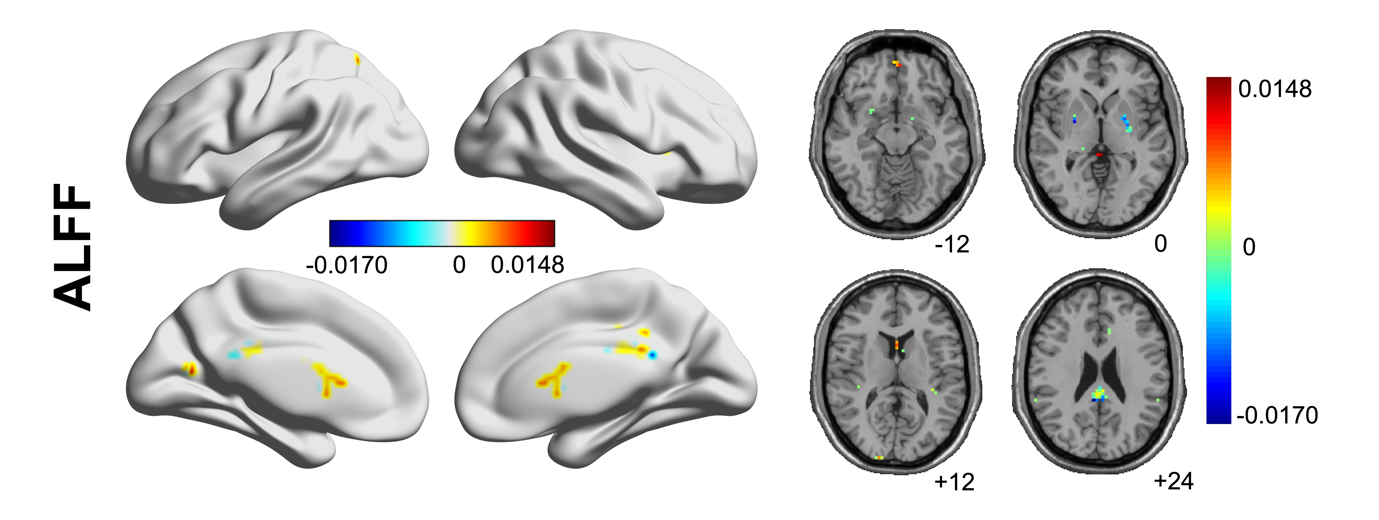


**SFigure 2d.** Validation results: discriminative voxel-wise weight of ALFF in the case that the data was preprocessed with motion scrubbing. The maps of GMV and FA were not shown as their extraction would not be influenced in this case. Warm color indicates positive weights, whilst cold color indicates negative weights. Darker color indicates larger absolute values of weights.


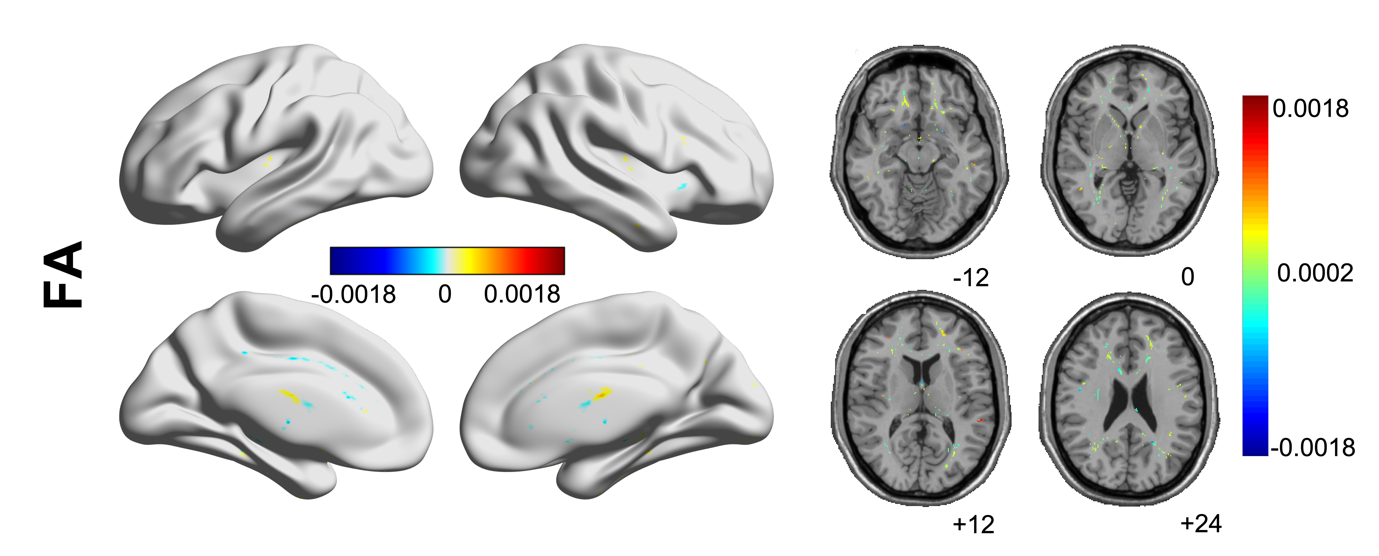


**SFigure 2e.** Validation results: discriminative voxel-wise weight of FA in the case that the data was preprocessed with TBSS. The maps of ALFF and GMV were not shown as their extraction would not be influenced in this case. Warm color indicates positive weights, whilst cold color indicates negative weights. Darker color indicates larger absolute values of weights.


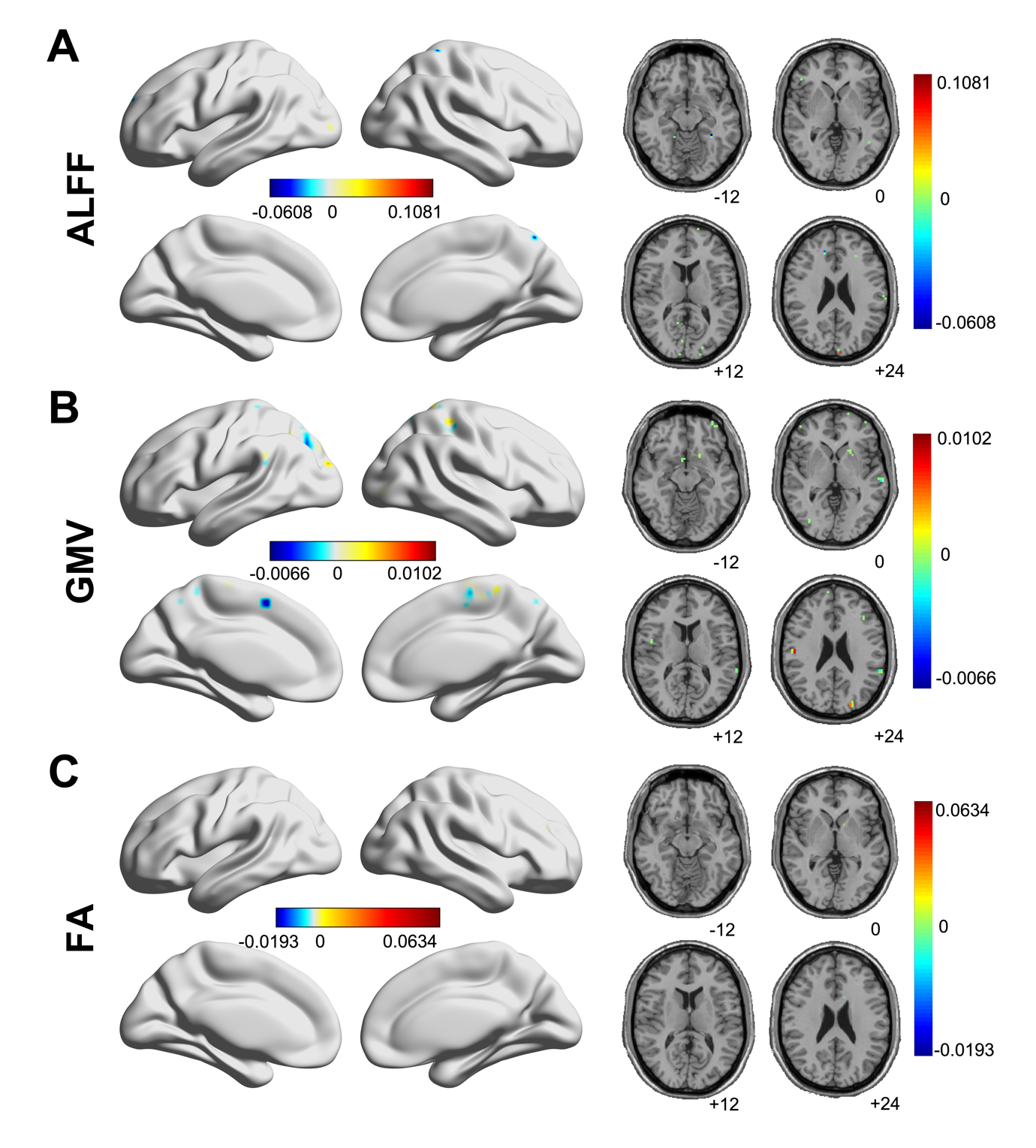


**SFigure 2f.** Validation results: discriminative voxel-wise weight of (A) ALFF, (B) GMV, and (C) FA in the case of emotional regulation prediction. In all the three maps, warm color indicates positive weights, whilst cold color indicates negative weights. Darker color indicates larger absolute values of weights.


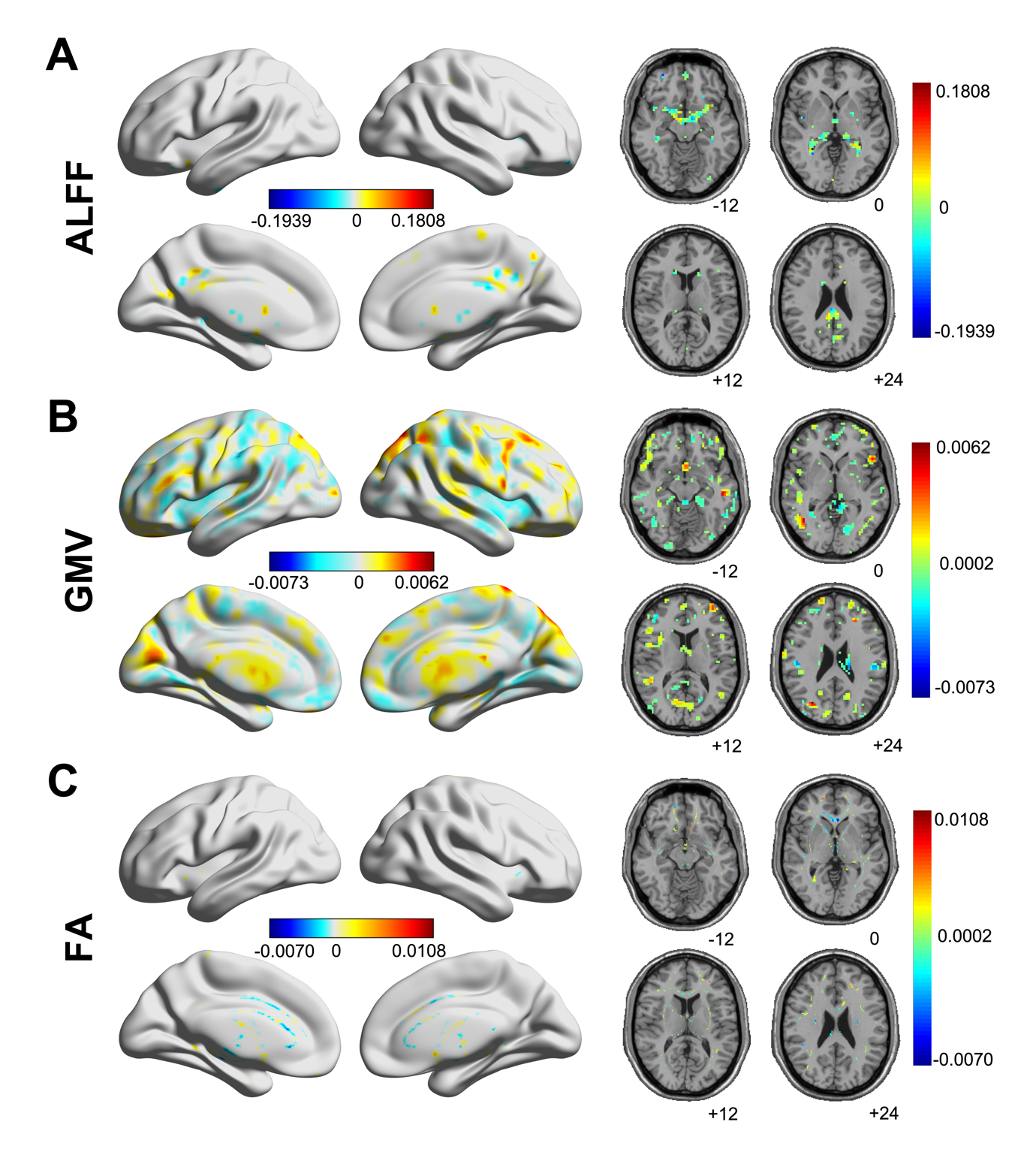


**SFigure 2g.** Validation results: discriminative voxel-wise weight of (A) ALFF, (B) GMV, and (C) FA in the case of fluid intelligence prediction. In all the three maps, warm color indicates positive weights, whilst cold color indicates negative weights. Darker color indicates larger absolute values of weights.


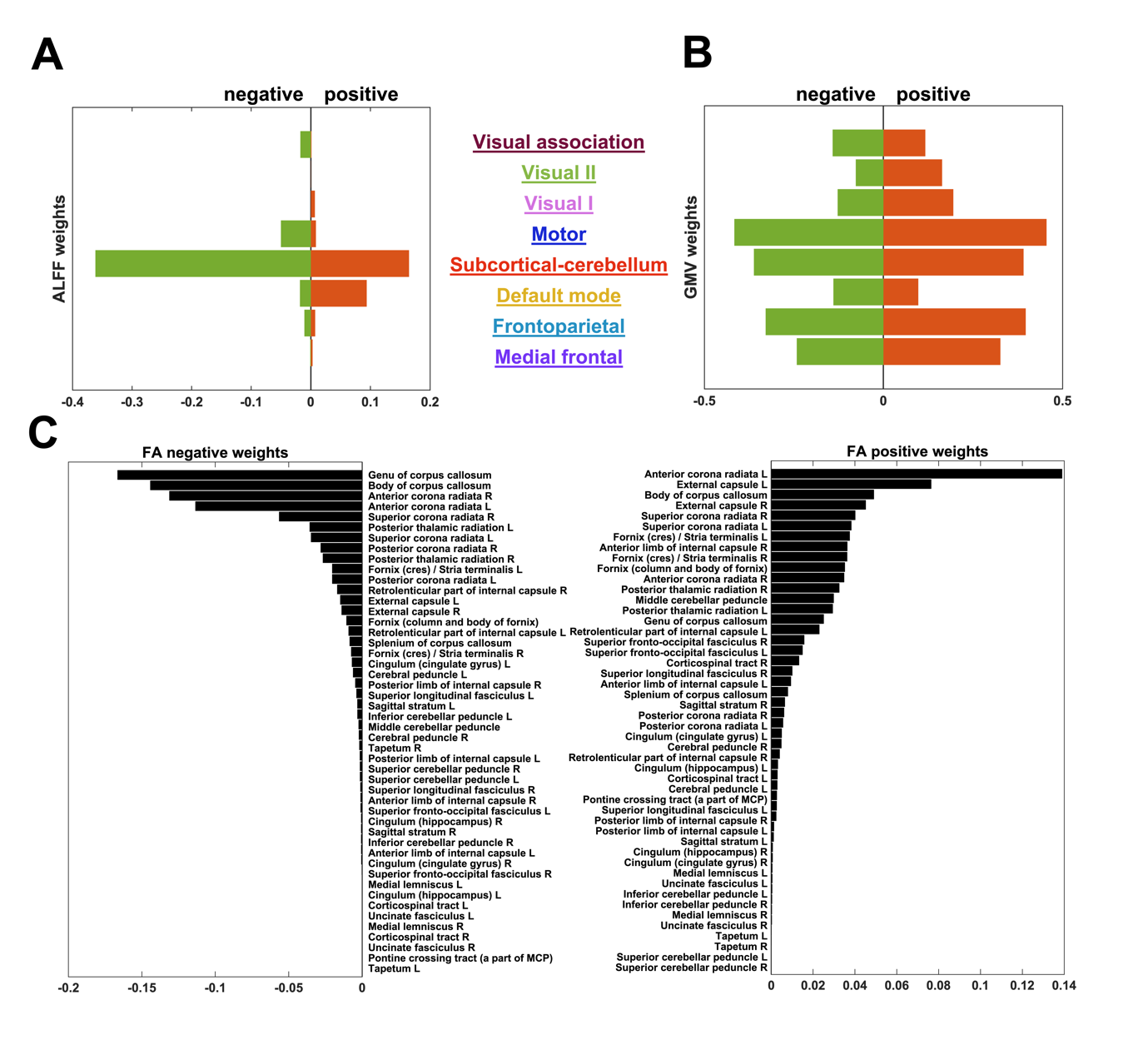


**SFigure 3a.** Validation results: respective sum of the positively and negatively contributive weights from (A) the eight networks of ALFF, (B) the eight networks of GMV, and (C) the 48 fiber tracts of FA in the case that the prediction model was built from 5-fold cross-validation. The network / fiber information was derived from the 268-node functional atlas (Shen et al., 2013; Finn et al., 2015) and JHU-ICBM DTI atlas (Mori et al., 2005), respectively.


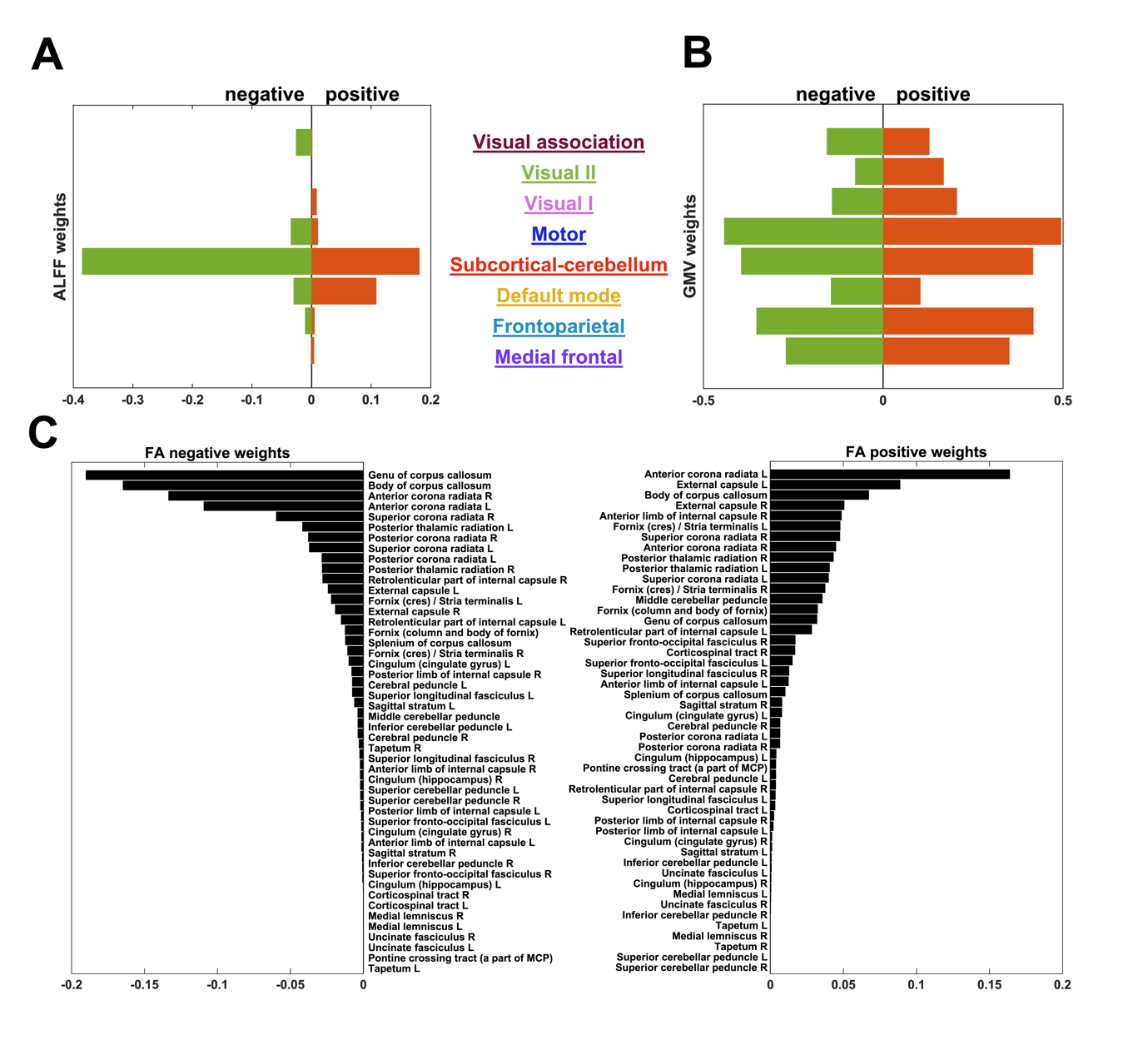


**SFigure 3b.** Validation results: respective sum of the positively and negatively contributive weights from (A) the eight networks of ALFF, (B) the eight networks of GMV, and (C) the 48 fiber tracts of FA in the case that the prediction model was built from 10-fold cross-validation. The network / fiber information was derived from the 268-node functional atlas (Shen et al., 2013; Finn et al., 2015) and JHU-ICBM DTI atlas (Mori et al., 2005), respectively.


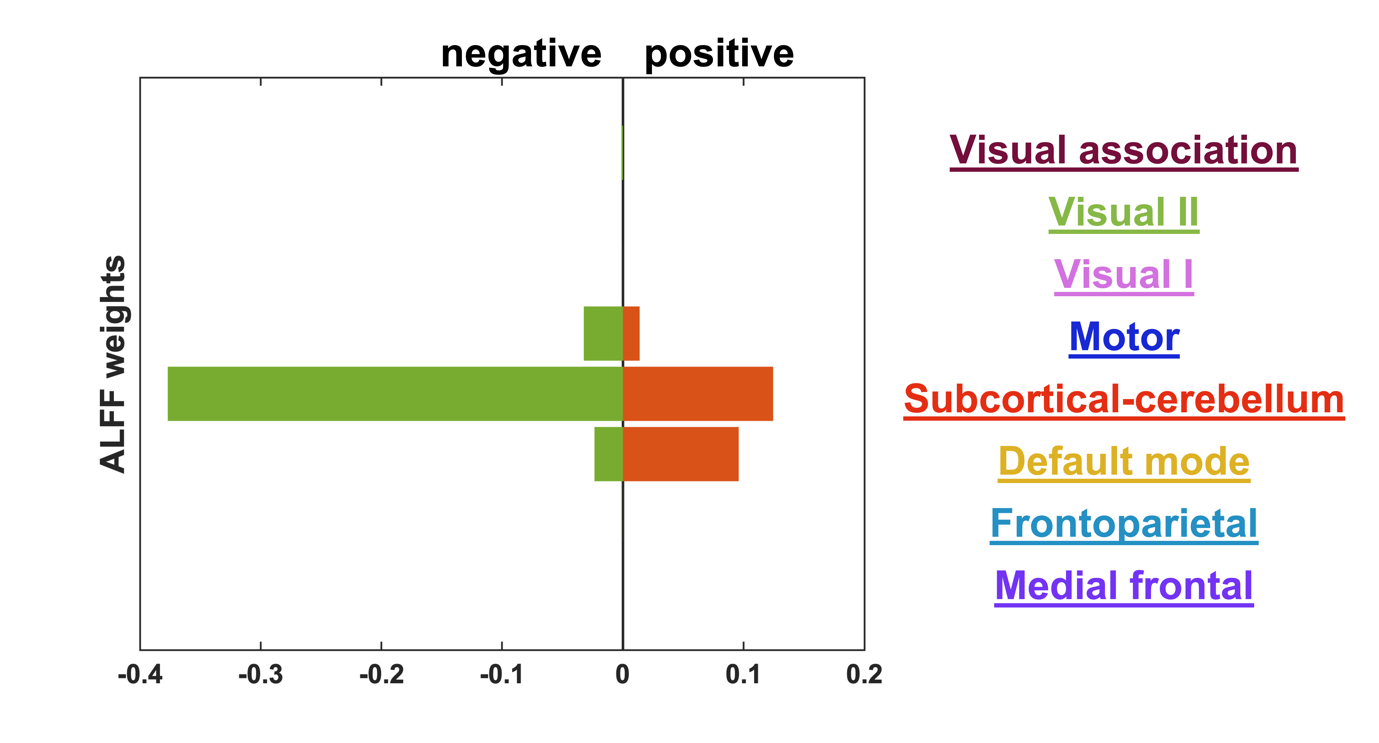


**SFigure 3c.** Validation results: respective sum of the positively and negatively contributive weights from the eight networks of ALFF in the case that the data was preprocessed without global signal removal. The network was derived from the 268-node functional atlas (Shen et al., 2013; Finn et al., 2015).


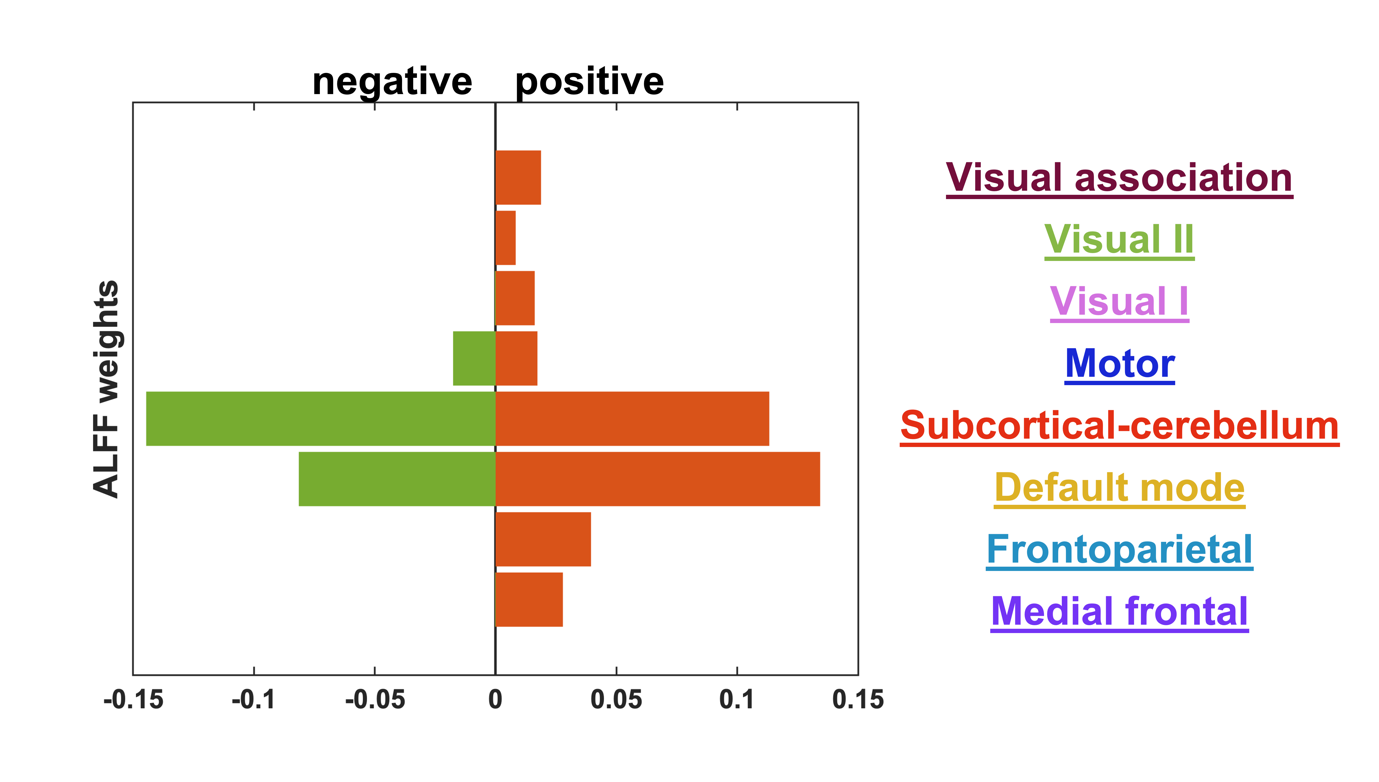


**SFigure 3d.** Validation results: respective sum of the positively and negatively contributive weights from the eight networks of ALFF in the case that the data was preprocessed with motion scrubbing. The network information was derived from the 268-node functional atlas (Shen et al., 2013; Finn et al., 2015).


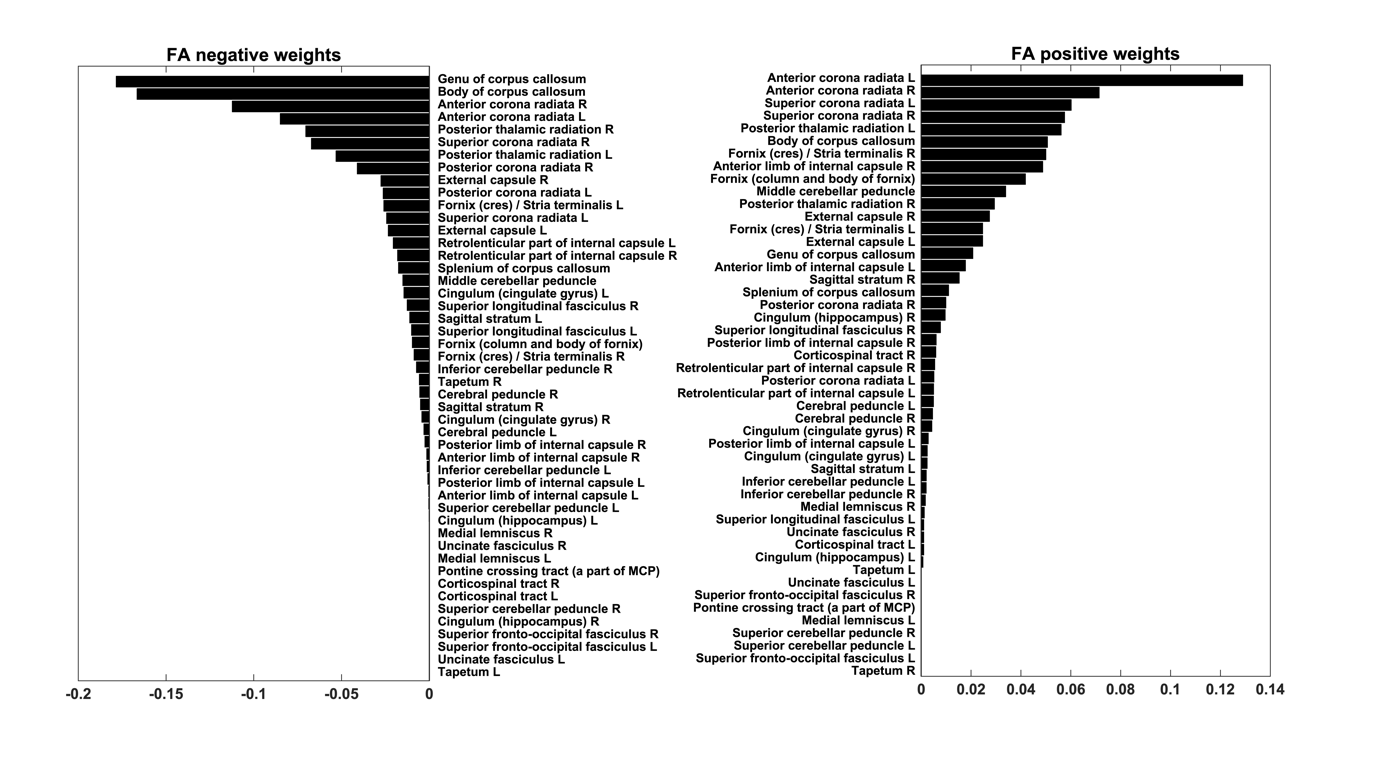


**SFigure 3e.** Validation results: respective sum of the positively and negatively contributive weights from the 48 fiber tracts of FA in the case that data was preprocessed with TBSS. The fiber information was derived from JHU-ICBM DTI atlas (Mori et al., 2005).


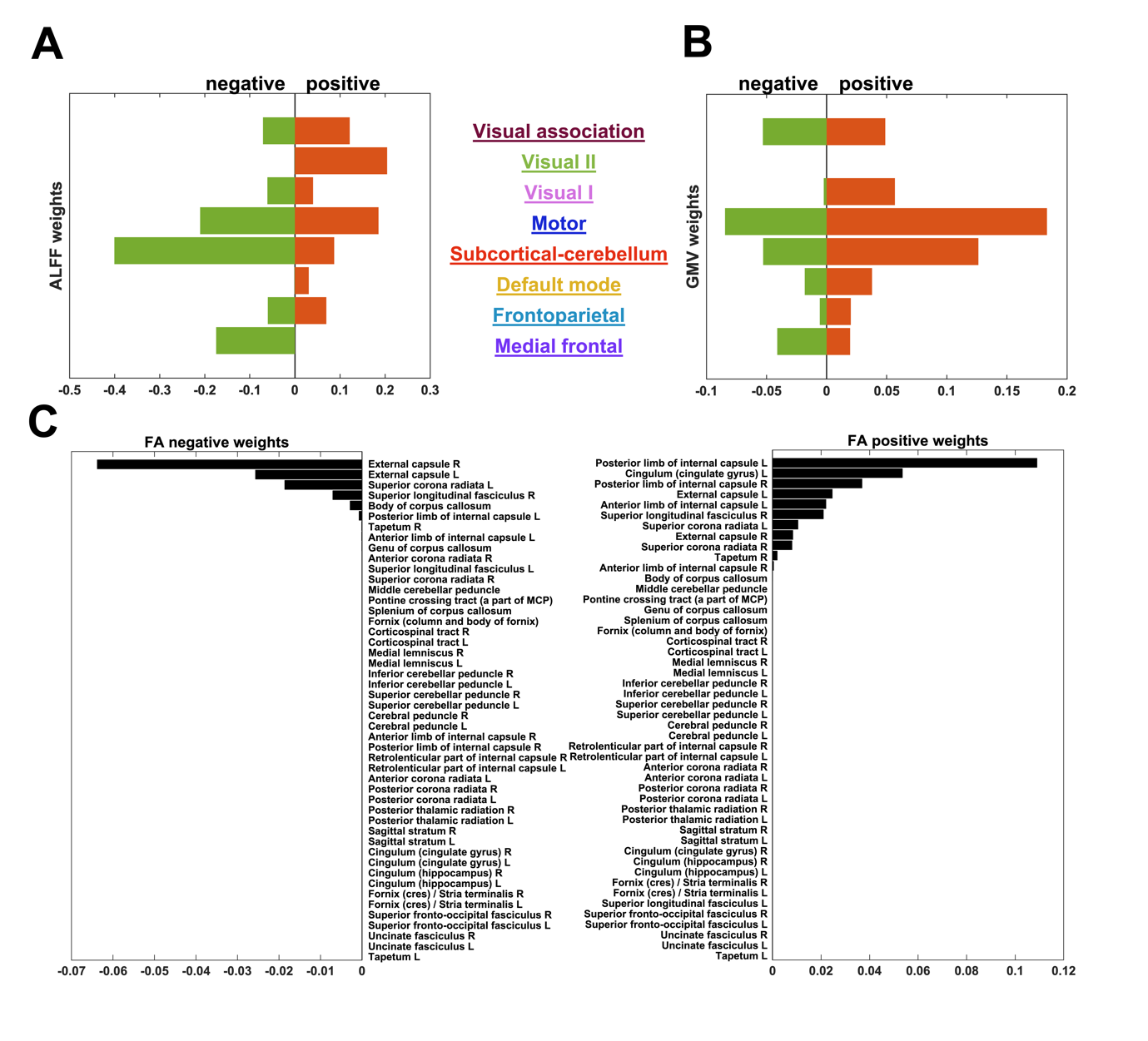


**SFigure 3f.** Validation results: respective sum of the positively and negatively contributive weights from (A) the eight networks of ALFF, (B) the eight networks of GMV, and (C) the 48 fiber tracts of FA in the case that the prediction model was built for emotional regulation prediction. The network / fiber information was derived from the 268-node functional atlas (Shen et al., 2013; Finn et al., 2015) and JHU-ICBM DTI atlas (Mori et al., 2005), respectively.


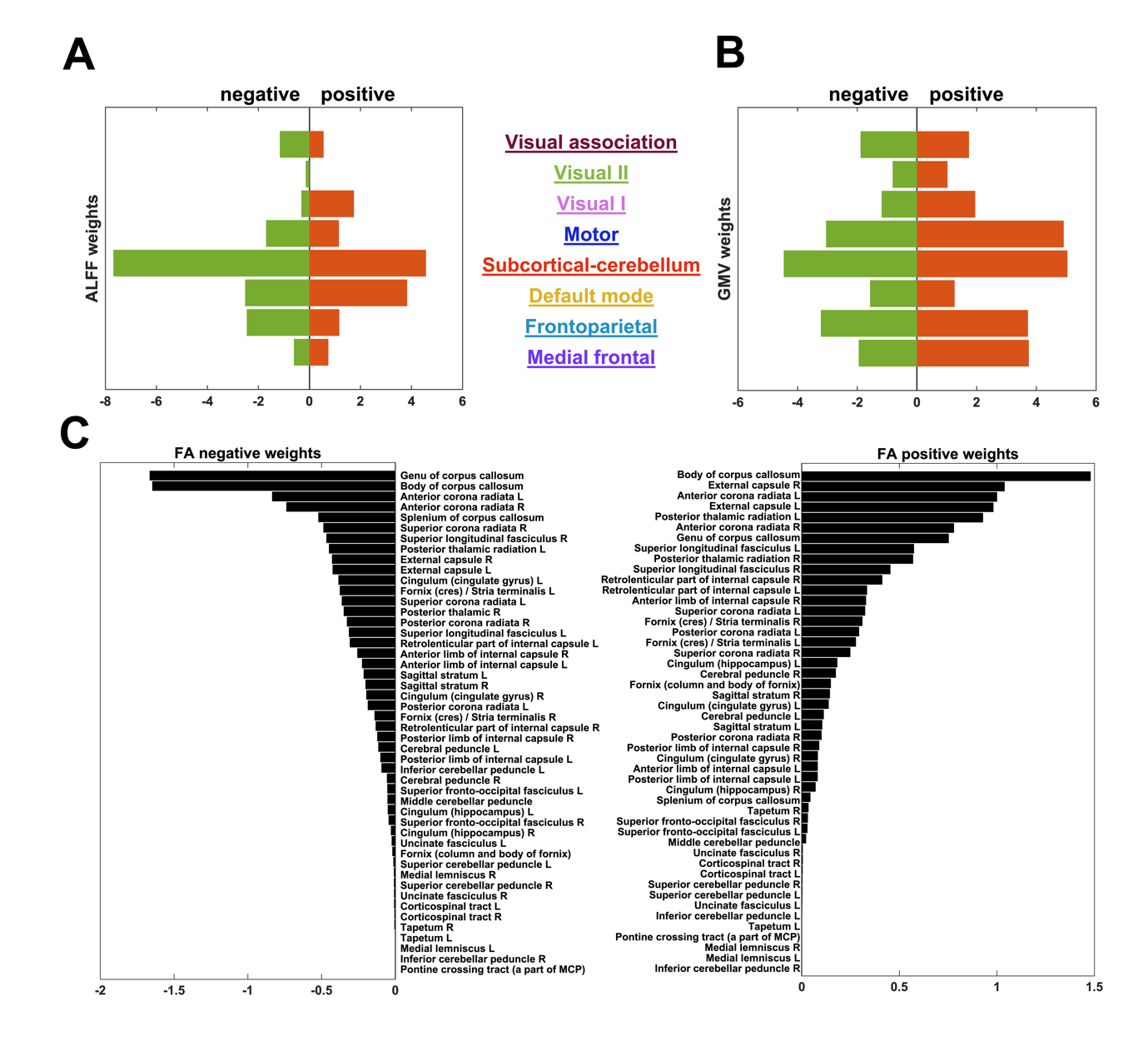


**SFigure 3g.** Validation results: respective sum of the positively and negatively contributive weights from (A) the eight networks of ALFF, (B) the eight networks of GMV, and (C) the 48 fiber tracts of FA in the case that the prediction model was built for fluid intelligence prediction. The network / fiber information was derived from the 268-node functional atlas (Shen et al., 2013; Finn et al., 2015) and JHU-ICBM DTI atlas (Mori et al., 2005), respectively.
